# Cell death mechanisms induced by gold nano-immunoconjugates-mediated photodynamic therapy against human oesophageal cancer stem cells

**DOI:** 10.3389/fimmu.2025.1585251

**Published:** 2025-09-16

**Authors:** Onyisi Christiana Didamson, Rahul Chandran, Heidi Abrahamse

**Affiliations:** Laser Research Centre, Faculty of Health Sciences, University of Johannesburg, Johannesburg, South Africa

**Keywords:** target therapy, oxidative stress, nanoparticles-mediated cell death, apoptosis, DNA damage response, cell cycle arrest, autophagy

## Abstract

**Background:**

The current conventional therapy for oesophageal cancer is unable to effectively eliminate oesophageal cancer cells as a result of cancer stem cells (CSCs). These CSCs are the main factors responsible for treatment failure and tumour relapse associated with the present conventional oesophageal cancer therapy. A nano-immunoconjugate-based photodynamic therapy (PDT) proposes a potential approach to eliminate these CSCs efficiently.

**Method:**

In this study, we examined the mode of cell death action induced by the nano-immunoconjugates (NIC) mediated PDT comprising aluminium phthalocyanine tetra sulfonic acid chloride (AlPcS_4_Cl), gold nanoparticles (AuNPs), and anti-CD271 antibody (AlPcS_4_Cl-AuNPs-anti-CD271) against human oesophageal CSCs *in vitro*. The oesophageal CSCs were treated with NIC-mediated PDT, and their impacts on cell viability, oxidative stress, mitochondrial membrane, efflux of cytochrome c protein, caspase 3/7 activity, and cell death mechanism were examined. We further evaluated the effects of the treatment on the various phases of the cell cycle, DNA damage response pathways, and autophagy.

**Results:**

Findings from this study showed that NIC-mediated PDT significantly inhibited the cell growth of oesophageal CSCs, promoted reactive oxygen species (ROS) production and mitochondrial-mediated apoptotic cell death through the alteration of mitochondrial membrane potential Δψm, high efflux of cytochrome c protein, high activity of caspase 3/7 protease, and early apoptosis. Moreover, NIC-mediated PDT triggered cell cycle checkpoint activity in the G0/G1 phase, stimulated DNA damage response by increased DNA double-strand breaks (DSB) and ATM (ataxia-telangiectasia mutated) upregulation, and activated an autophagy action.

**Conclusion:**

The outcomes from this study showed the anticancer efficiency of gold nano-immunoconjugate-based PDT against human oesophageal CSCs. Overall, this study provides a rationale for gold nano-immunoconjugate-based PDT for a promising therapeutic application in the clinical treatment of oesophageal cancer.

## 1 Introduction

Oesophageal cancer is a tumour of the digestive system characterised by no symptoms, late diagnosis, metastasis, relapse, poor prognosis, and high mortality rate. A recent report ranked oesophageal cancer as the eleventh cause of cancer-related disease and the seventh cause of cancer-promoted death globally (1, 2). The asymptomatic feature of oesophageal cancer is a significant threat to the management of this malignancy. Advances in the healthcare system have made tremendous progress in the management of oesophageal cancer, and the treatment measures include surgery, chemotherapy, radiotherapy, photodynamic therapy, and molecular targeted therapy (3–6). Though the present conventional therapy for oesophageal cancer has decreased the burden of this malignancy to some extent, resistance to treatments and disease progression remain huge problems to overcome. Studies have shown that cancer stem cells (CSCs) are the main factors responsible for treatment failure and tumour relapse associated with the present conventional therapy for oesophageal cancer (7–9). Cancer stem cells are a subset of cells in the tumour with cancer initiation capacity, self-renewal potential, and the ability to differentiate into multiple lineages. Various studies have reported the prognostic importance of CSCs in oesophageal cancer, and targeting CSC markers has attracted much attention (10, 11). Different CSC markers such as CD271(p75NFR), CD44, CD90, CD133, ALDH1, and many others have been identified in oesophageal cancer (12–15). CD271, known as p75NTR (p75 neurotrophin receptor), is a neurotrophin receptor that belongs to the superfamily of the tumour necrosis factor receptor and plays a vital role in neuronal survival, cellular proliferation, migration, and invasion in various tumour cells (16–19). Studies have demonstrated that CD271+ oesophageal cancer cells possess self-renewal, drug resistance capacity, and are mitotically quiescent (16, 20, 21). Notably, high expression of CD271 is linked with poor prognosis in oesophageal cancer (16, 20) as well as in other cancers such as bladder cancer (18), laryngocarcinoma (22), and head and neck squamous cell carcinoma (17). Therefore, targeting oesophageal CSCs markers using an alternative approach like photodynamic therapy (PDT) offers a promising strategy to overcome treatment failure.

Photodynamic therapy is a less invasive light-activated therapeutic measure approved by the Food and Drug Administration (FDA) for treating different diseases, including cancer (23). Basically, PDT entails the administration of a photoactive agent referred to as a photosensitizer which accumulates within tumours and subsequent exposure to irradiation at a particular wavelength of light in the presence of molecular oxygen, results in cascades of chemical reactions that lead to the generation of singlet oxygen/reactive oxygen species (ROS) and cellular damage (23, 24). Photodynamic therapy is an effective therapeutic approach that eliminates cancer cells while sparing non-tumour cells. The potential application of PDT to eradicate CSCs has recently attracted much interest (25–28). The therapeutic effectiveness of PDT is reliant on the light dose, presence of molecular oxygen, and the photosensitizer used (29). Various photosensitizers have been used for the treatment of oesophageal cancer (30, 31), and aluminium (III) phthalocyanine chloride tetra sulphonic acid (AlPcS_4_Cl) has attracted much attention due to its photophysical and photochemical features, especially its hydrophilicity and high absorption that allows in-depth tissue penetration thereby promoting efficient treatment of deep-seated cancers (32–34). However, in spite of the many advantages of AlPcS_4_Cl, the uneven delivery and lack of tumour-specificity of this photosensitizer in targeting cancer and CSCs pose a significant limitation. The uneven delivery/biodistribution of most conventional photosensitizers including AlPcS_4_Cl results in non-tumour selectivity and low tumour cell uptake, thereby limiting the efficacy of PDT (35–37).

To address this challenge, PDT has witnessed a paradigm shift in recent years through the advent of nanotechnology. This advancement has ushered in new ways to enhance PDT effects by enhancing the photochemical reaction with adequate reactive oxygen species (ROS) generation, improving drug delivery/tumour selective targeting, and designing/modulating biomolecules/photosensitizers by incorporating nanoparticles (3, 38). Nanoparticles possess unique properties such as excellent biocompatibility, versatile surface chemistry, superior optical features for imaging, chemical inactivity, and distinct shapes and sizes. Nanoparticles’ surface chemistry/modification is exploited to attach site-specific biomolecules (such as peptides, antibodies, biotin, folate), imaging labels, and treatment agents with various applications, especially as a delivery system. Nanoparticles could be delivered to tumour cells as drug nano-cargos through passive or active targeting strategies (37–39). The emergence of nano-immuno-conjugates comprising gold nanoparticles (AuNPs), antibody, and a photosensitizer is a potential strategy to enhance the delivery and tumour-specific targeting of PDT in cancer and CSCs (27, 38, 40, 41). The use of AuNPs for active targeting enables surface modulation with different biomolecules with high affinity for tumour cells. The targeting biomolecules control the selective attachment of nanoparticles to the receptor/proteins expressed on tumour cells and CSCs, thereby promoting the internalisation of the nano-drug (38, 42). Furthermore, nanoparticles for active targeting can be designed to deliver a drug to the CSC tumour microenvironment compared to a free drug. This can be accomplished by designing nanocarriers capable of targeting the CSC niche, such as the signalling molecules, CSC marker, and the various components of the CSC niche (38, 43). To overcome the challenges whereby the targeted receptor of interest is expressed in normal tissue and tumour cells, an effective drug delivery system that transports the antibody selectively to the tumour cell is crucial (44).

Several investigations have evaluated the use of AuNP for active targeted PDT on different tumour types, demonstrating the efficiency of this approach. For instance, AuNPs, C11Pc (photosensitizers), and the epidermal growth factor receptor (EGFR) peptide AEYLR were used to attack EGFR receptors in lung tumours in an *in vitro* model (45). The combined use of transferrin, AuNP modified with poly (styrene-altmaleic acid) (PSMA), and methylene blue was employed in an *in vitro* model to treat cervical malignant cells (46). Furthermore, in a preclinical study, breast cancer cells were treated with phthalocyanine, anti-HER2 antibody, and a PEGylated AuNP to target HER2-expressing breast cancer cells (32) selectively. In another instance, prostate-specific membrane antigen (PSMA) peptide, AuNP, and silicon phthalocyanine were employed to attack prostate cancer PSMA receptors using preclinical models (20). Furthermore, in another *in vitro* study, anti-CD133 antibodies, AuNPs, and aluminium phthalocyanine tetra sulfuric chloride were used to treat CD133-positive lung cancer cells (41). The use of AuNPs for active targeted PDT has proven effective and selective for inducing cell death and eliminating hard-to-kill cancer cells such as CSCs. However, studies using AuNPs-antibody conjugate for active targeted PDT against oesophageal CSCs and possible cell death mechanisms are lacking. Therefore, nano-immuno-conjugate mediated PDT consisting of AuNPs, CD271 antibodies coupled with AlPcS_4_Cl for enhanced PDT against oesophageal CSC is of paramount importance for the elimination of oesophageal cancer.

In this study, a multi-component gold nano-immuno-conjugate (NIC) comprising AlPcS_4_Cl, AuNPs, and anti-CD271 antibody previously synthesized and characterized was employed (27) to examine the enhanced therapeutic potentials of NIC-based PDT in oesophageal CSCs. In this study, the CD271 antibodies were leveraged as a targeting molecule against oesophageal CSCs. Studies have shown that CD271 surface antigens are highly expressed in oesophageal cancer, and they play a crucial role in promoting drug resistance and maintaining the stemness features of oesophageal cancer (10, 47, 48). Hence, the CD271 antibodies are essential for specific active targeting of oesophageal CSCs, while the AuNPs served as a nanocarrier for enhancing the delivery/cellular uptake of the photosensitizer. Therefore, the *in vitro* therapeutic potentials and the possible cell death mechanism mediated by NIC-based PDT in oesophageal CSCs were evaluated to provide insight for its translational/clinical applications.

## 2 Materials and methods

### 2.1 Cell culture and CSC isolation

A commercial HKESC-1 (RRID: CVCL_D568) oesophageal cancer cell line (Cellonex, Johannesburg, South Africa) was utilised for this study. Prior to oesophageal CSCs isolation the HKESC-1 cells were cultured in DMEM (Dulbecco’s modified Eagle’s medium) (D5796, Sigma-Aldrich) and supplemented with 1 mM sodium pyruvate, 0.5% amphotericin B (A2411, Sigma), 0.5% penicillin-streptomycin (P078, Sigma) and 10% foetal bovine serum (FBS) (10082147, Gibco). The CSCs were isolated from the HKESC-1 cells utilising the CD44, CD90, and CD271 Microbeads Kits (130-095-194/130-096-253/130-099-023, Miltenyi Biotec) via a magnetic separation method. The isolated oesophageal CSCs were cultured and maintained in DMEM-F12 (D8062, Sigma-Aldrich) medium supplemented with 0.5% FBS, 20 ng/mL epidermal growth factor (EGF) (PHG0315, Gibco), 20 ng/mL basic fibroblast growth factor (bFGF) (PHG0266, Gibco), 0.5% penicillin/streptomycin and 0.5% amphotericin B. The oesophageal CSCs were cultivated in T75 Corning^®^ flasks (CLS430641, Sigma) at a cell concentration of 1.5 × 10^6^ cells/mL and maintained in an 85% humidified incubator at 37°C with 5% carbon dioxide (CO_2_) to about 95% confluence. For the downstream experiment, the cells were harvested and cultivated at a concentration of 5 × 10^5^ cells in a 35 mm diameter Corning^®^ cell culture plate (CLS430165, Sigma) containing 2 mL of supplemented DMEM-F12 medium.

### 2.2 Immunofluorescence characterisation of oesophageal CSCs

The oesophageal CSCs were characterised using the indirect immunofluorescence technique, which utilises fluorescence imaging for identification. The CSCs were cultivated and kept in the incubator for 24 hours to permit adequate attachment. Following a 24-hour incubation period, the cells were rinsed two times using ice-cold buffer (PBS/BSA/azide) and fixed for 15 minutes with 1 mL of 4% paraformaldehyde. The cells were rinsed with an ice-cold buffer, and the supernatant was removed. A 1 mL of blocking buffer (10% (w/v) BSA/PBS) was pipetted and placed on ice for half an hour. The cells were rinsed with PBS, and 100 µL of diluted (in 1x PBS 1:1000) primary mouse antibodies (Anti-CD44 (MA515462), Anti-CD90 (14–0909–82), and Anti-271 (MA515574)) was added to the cells. The cells were placed on ice for half an hour, washed thrice with ice-cold buffer, and tagged with 100 µL of diluted secondary antibody (FITC Goat anti-Mouse diluted in 1x PBS 1:1000) for half an hour. Afterwards, the cells were rinsed and labelled with a nuclear dye (DAPI) for 10 minutes, and the coverslips were gently fitted on glass slides using Fluoromount™. The slides were examined with a Zeiss Axio Z1 observer (Zeiss, Johannesburg, South Africa) inverted fluorescence microscope. The mean fluorescence intensity (MFI) of the images was analysed using ZEN 3.1 software (Carl Zeiss, Germany).

### 2.3 Hoechst characterisation of oesophageal CSCs

The stemness features of the isolated cells were analysed using the Hoechst 33342 dye efflux approach. This assay is based on the mechanism that CSCs have high expression of ABCG2 transporter molecules, which are known to expel certain dyes, such as Hoechst 33342 dye. Cells with stemness features efflux the Hoechst 33342 dye, displaying low fluorescence when viewed with flow cytometry and fluorescence microscopy (49). Briefly, the cells were cultivated and kept in an incubator overnight at a humidity of 95%, 5% CO_2_, and 37°C. Following incubation, the medium was discarded and rinsed with 1x PBS. The cells were labelled with 200 µL of 5µg/mL Hoechst 33342 dye (H1399, Invitrogen) and incubated at 37°C for 20 minutes. Thereafter, cells were rinsed thrice in 1x PBS to expel any free dye. Finally, the cells were viewed, and the image was taken with a Carl Zeiss Axio Z1 microscope using the 359Ex/461Em filters. The images were examined, and the fluorescence signal was measured using the ZEN version 3.1 software (Carl Zeiss, Germany).

### 2.4 Photosensitizer and nano-immune conjugates preparation

The nano-immuno-conjugates (NIC) used in this study were synthesized using a chemical reaction and characterized as previously described (27). The NIC consists of three multi-components: AlPcS_4_Cl (AlPcS-834, Frontier Scientific), spherical gold nanoparticle (AuNP-PEG-COOH) (3 mg/mL, 10 nm diameter, 765457, Sigma-Aldrich), and anti-CD271 antibody (NBP2-80874, NOVUSBIO). Briefly, 4.5 mg of AlPcS_4_Cl was dissolved in 5 mL of purified deionised water to obtain a stock concentration of 1 mM. The stock concentration of AlPcS_4_Cl was kept at 4°C in the dark. The preparation of AlPcS_4_Cl-gold nanoparticles (AuNPs) was achieved by electrostatic interaction. Briefly, AlPcS_4_Cl-AuNPs conjugates were prepared in a 1:2 ratio at a concentration of 20 µg/ml (AlPcS_4_Cl) and 40 µg/ml (AuNPs). The mixture was continuously spun for 24 hours, purified via centrifugation, reconstituted in PBS (1x), and stored in the dark at 4°C. The NIC was formulated by chemical reaction and physical adsorption as previously described (27, 40). Briefly, 60 mg of N-(3-Dimethylaminopropyl)-N′-ethylcarbodiimide hydrochloride (EDC) (Sigma Aldrich: E1769) and 72 mg of N-Hydroxysulfosuccinimide sodium salt (sulfo-NHS) (Sigma Aldrich: 56485) were dissolved in 500 μL of 2-(N-morpholino) ethane sulfonic acid (MES) 10 mM, pH 5.5 (Sigma Aldrich: M3671). The solutions were added to obtain a final concentration mixture of 30 mg/mL and 36 mg/mL for EDC and sulfo-NHS, respectively. An equal volume of AuNPs (100μL, 20 μg/mL) and EDC/NHS solution (100 μL) was added into a 2 mL centrifuge tube and incubated at 25 °C for 1 hour, then at 30 minutes. The mixture was washed in (1 x PBS pH 7.4, and 0.05% TWEEN^®^ 20 (w/v)), centrifuged for 1 hour at 1,600 x g, and the supernatant removed.

Thereafter, anti-CD271 antibody (NBP2-80874, NOVUSBIO)(100μL, 20 μg/ml) was added, appropriately mixed, and incubated at room temperature for 120 minutes. The solution was purified by centrifuging at 1,600 x g for 60 minutes. The supernatant was removed. Conjugate storage buffer (20 mM Tris (pH 8.0), 150 mM NaCl, 1% BSA (w/v))(500 μL) was added. Finally, AlPcS_4_Cl (500 μL, 20 μM) was added to the complex (AuNP-PEG-COOH-Anti-CD27), and the mixture was continuously spun by a vortex (Vortex-Genie 2, Scientific Industries) for 24 hours at 300 RPM. The newly formed AlPcS_4_Cl-AuNP-Anti-CD271 conjugate, known as nano-immuno-conjugates (NIC), underwent purification by centrifugation, dissolved in PBS (1x, 1mL), and stored at 4°C until ready for use. The synthesized NIC was characterized and confirmed in our previous studies (27).

### 2.5 Characterization of nano-immuno-conjugates

#### 2.5.1 Transmission electron microscopy

Transmission electron microscopy (TEM) analysis was conducted to determine the shape and size of AuNPs and NIC using the JEM-2100 Transmission Electron Microscope (JEOL Ltd., Tokyo, Japan). The AuNPs and NIC solutions were added onto copper TEM grids (carbon-coated 200 Mesh) and allowed to dry. The grids were placed into the TEM sample carrier, examined, and micrographs captured.

#### 2.5.2 Ultraviolet-visible spectroscopy

The UV-Vis spectroscopy analysis was conducted to determine the optical properties of AlPcS_4_Cl, AuNPs, Anti-CD271 antibody, and NIC using a spectrophotometer (Jenway, GENOVA NANO, 67912). The spectral analysis of AlPcS_4_Cl, AuNPs, Anti-CD271 antibody, and NIC was evaluated at a resolution of 2 nm through a wavelength region of 200–800 nm.

### 2.6 Cellular uptake

The oesophageal CSCs were cultured, and after overnight incubation, the cell culture medium was removed and incubated with fresh culture media containing 20 µM AlPcS_4_Cl and 20 µG/mL NIC. The cells were incubated for 4 hours at 37°C, washed with PBS, and fixed with 4% paraformaldehyde for 15 min. Afterwards, the cells were rinsed and labelled with 300nM DAPI for 10 minutes, and the coverslips were carefully placed on glass slides using Fluoromount™. The Zeiss Axio Z1 observer (Zeiss, Johannesburg, South Africa) inverted fluorescence microscope was used to examine the slides. The mean fluorescence intensity (MFI) of images was analysed using ZEN 3.1 software (Carl Zeiss, Germany).

### 2.7 *In vitro* photodynamic treatment

Cells were cultured and kept in a humidified incubator overnight for this experiment. Thereafter, the culture medium was discarded, washed with HBSS, and refreshed with a growth medium containing the IC_50_ of AlPcS_4_Cl (5 µM), AlPcS_4_Cl-AuNPs (3 µg/mL), and NIC (1.3 µg/mL) in triplicate as previously obtained (27). The experimental cells were categorised into irradiated and non-irradiated groups. As previously described, the culture dishes were protected with aluminium foil and placed in the incubator for 4 hours (27). Following incubation, the cells were rinsed thrice with HBSS. Finally, the cells received 1 mL PBS and irradiated using a semiconductor diode laser (Arroyo, National Laser Centre, South Africa) at a fluency of 5 J/cm^2^, 97 mW power output, and 673.2 nm wavelength. A fluency of 5 J/cm^2^ was chosen based on previous optimized fluency using the same photosensitizer (34). The medium was replaced with PBS to prevent laser energy absorption by the medium. After irradiation, the cells were restored to a DMEM-12 culture medium and then incubated for 24 hours. The cell death mechanism induced by AlPcS_4_Cl, AlPcS_4_Cl-AuNPs, and NIC was evaluated.

### 2.8 Cell viability

The luminescence cell viability assay from Promega (G968) was employed to determine the effects of the AlPcS_4_Cl, AlPcS_4_Cl-AuNPs, and NIC on the viability of CSCs. To analyse the cell viability, 50 μL each of harvested cells and CellTiter-Glo^®^ 3D reagent was pipetted into an opaque 96-well plate and kept at 25°C for 10 minutes. Finally, the cell viability was analysed by measuring the ATP luminescence produced using the Perkin Elmer VICTOR Nivo™ plate reader.

### 2.9 Reactive oxygen species production

To determine the level of cellular reactive oxygen species (ROS) produced, ROS assay from Abcam (ab113851) using the fluorescent probe 2′,7′-dichlorofluorescein diacetate (DCFDA/H2DCFD) was utilised as previously described (33). The oesophageal CSCs were cultured in 96-well tissue culture plates at a concentration of 1.0 x 10^4^ cells/well containing 100 μL culture medium. Twenty-four hours after PDT, the cells were exposed to 5 μM of DCFDA/H2DCFDA and kept at 37°C for 30 minutes of incubation. Following incubation, the cells were moved into a 96-well black plate and analysed using the PerkinElmer VICTOR Nivo™ plate reader with a 485 nm/538 nm excitation/emission filter. The amount of ROS formed in the experimental cells was compared with that in the control cells.

### 2.10 Mitochondrial membrane assessment

The rhodamine-123 efflux fluorimetry analysis using rhodamine-123 dye (ab275545) from Abcam was utilised to evaluate the mitochondrial membrane potential (Δψm) in oesophageal CSCs as earlier reported (33). Following 24 hours post-PDT application, the cells were detached, washed two times in 1x PBS, and the supernatants were eliminated. Thereafter, 100 µL of 1x PBS containing 25 μM of rhodamine-123 was loaded into the cells. The cells were kept at 25°C for 15 minutes, protected from light. The cells were rinsed two times, the supernatant was expelled, and then 400 μL of 1x PBS was dispensed and adequately mixed. Thereafter, 100 μL of the cells were transferred into a 96-well black plate and incubated for 30 minutes in the dark. The PerkinElmer VICTOR Nivo™ plate reader was used to capture the fluorescence signals using 505/535 nm excitation/emission filters.

### 2.11 Cytochrome c measurement

The efflux of cytochrome c protein in the cell lysate was measured as previously described (33) using the cytochrome c ELISA assay (BMS263) obtained from Invitrogen. Three hours after PDT, 1mL lysis buffer was added to 1.5 x 10^6^ cells and kept for 60 minutes at 25 °C. The cells underwent centrifugation for 15 minutes at 200 x g, and the lysate was diluted in 1:50 of 1x assay buffer. The microwell strips received 100 μL of the diluted samples, and blank wells, which serve as a control, received 100 μL of assay buffer. All the microwell strips received biotin anti-cytochrome c antibody (50 μL, 1:100 dilution). The microwell strips were sealed with an adhesive sheet and allowed to stand for two hours at 25°C. Afterwards, the microwell strips were rinsed thrice with 500 μL wash buffer using a microplate washer, and streptavidin-HRP secondary antibody (100 μL, 1:200 dilution) was pipetted into the microwell strips. The wells were sealed with an adhesive sheet and kept at 25°C for one hour. The microwell strips were rinsed, and tetramethyl-benzidine (TMB) substrate (100 μL) was pipetted and kept for 10 minutes at 25°C. After incubation, stop solution (100 μL) was added to all the microwell strips. The absorbance at 450 nm wavelength was measured with a microplate reader (PerkinElmer VICTOR Nivo™).

### 2.12 Cell death evaluation

The Annexin V-FITC/PI assay (556570, BD Pharmingen™) was employed to evaluate the cell death induction. In summary, the CSCs were harvested, washed, and Annexin V-FITC binding buffer (500 μL) was added to form a cell suspension. Thereafter, the cells were stained with annexin V-FITC (5 μL) and propidium iodide (PI) (5 μL) and allowed to stand for 15 minutes at 25°C. The CSCs were analysed using flow cytometry (BD flow cytometer Accuri™ C6).

### 2.13 Caspase 3/7 quantification

The Muse^®^ caspase 3/7 assay (MCH100108, Luminex) was used to examine the caspase 3/7 activity in compliance with the manufacturer’s user guides. Following three hours post-PDT treatment, the cells were detached, and 1 x 10^5^ to 5 x 10^6^ cells were suspended in 1 mL of 1x assay buffer BA. The caspase 3/7 working reagent was obtained by diluting the Muse^®^ caspase 3/7 reagent in a ratio of 1:8 with 1x PBS. Afterwards, the cells were suspended in 1x assay buffer BA (50 μL), and the Muse Caspase-3/7 working reagent (5 μL) was added to 1.5 mL tubes and mixed properly. The tubes were kept in a 37°C incubator for 30 minutes. After incubation, 150 μL of the Muse Caspase 7-AAD working solution was loaded into each tube. The tubes were mixed properly and allowed to stand for 5 minutes at 25°C. The Muse^®^ Cell Analyser (Guava, Millipore) was used to measure the caspase 3/7 activity in the samples.

### 2.14 Cell cycle evaluation

The cell cycle assay (MCH100106, Millipore) was utilised to examine the cell cycle distribution. After 24 hours of PDT application, the CSCs were detached, 1.0 x 10^6^ cells were fixed in 70% ethanol and placed in a -20°C refrigerator for 3 hours. The cells were washed afterwards, and the Muse^®^ cell cycle reagent (200 μL) was added and kept at 25°C for 30 minutes. The cell cycle was analysed using the Muse^®^ Cell Analyser (Guava, Millipore).

### 2.15 DNA damage assessment

The DNA damage assay from Luminex (MCH200107) was utilised to analyse the DNA damage response. Briefly, 24-hour post-PDT exposure, oesophageal CSCs were harvested, and 1 x 10^5^ cells were suspended in 1 × assay buffer (50 μL), and a fixation buffer of equal volume (50 μL) was added and kept at 2-4°C for 10 minutes. The CSCs were washed and then permeabilised with 100 μL of 1x permeabilization buffer for 10 minutes at 2-4°C. The CSCs were labelled with an antibody cocktail (100 μL)(comprising anti-phospho-Histone H2A.X and anti-phospho-ATM antibodies) and incubated for half an hour. The cells were rinsed and resuspended in 1x assay buffer (200 μL). The samples were analysed using the Muse^®^ Cell Analyzer (Guava, Millipore).

### 2.16 Measurement of autophagy

The Autophagy LC3 antibody assay (MCH200109) from Luminex) was employed to measure the formation of autophagosome, which is an indication of autophagy. Prior to analysis, the CSCs were cultivated in a 96-well culture plate at a seeding concentration of 4.0 x 10^4^ cells/well for 24 hours and treated as described in section 2.4. Twenty-four hours after PDT, the cells were incubated with autophagy reagent A (200 μL) and placed at 37°C for 4 hours. The cells were detached, rinsed, and labelled with Anti-LC3 antibody (100 μL) diluted working solution and placed at 4°C for half an hour. The CSCs were rinsed, and 200 μL 1x assay buffer volume was added, carefully mixed, and analysed using the Muse^®^ Cell Analyzer (Guava, Millipore). Autophagy induction ratio (AIR) values ≥1 indicate autophagy induction, and values ≤ indicate no induction.

### 2.17 Statistical evaluation

Experimental values used in this study were displayed as mean ± standard error (SEM). Experiments were carried out at least twice and in triplicate (n=3). All statistical evaluations were done with GraphPad Prism version 5 (GraphPad Software Inc., CA, USA). ANOVA (One‐way analysis of variance) and the Tukey test were utilised to examine the statistical significance among the various sample groups. A *P*‐value < 0.05 was defined as significant.

## 3 Results

### 3.1 Immunofluorescence characterisation of oesophageal CSCs

**Figure 1:**
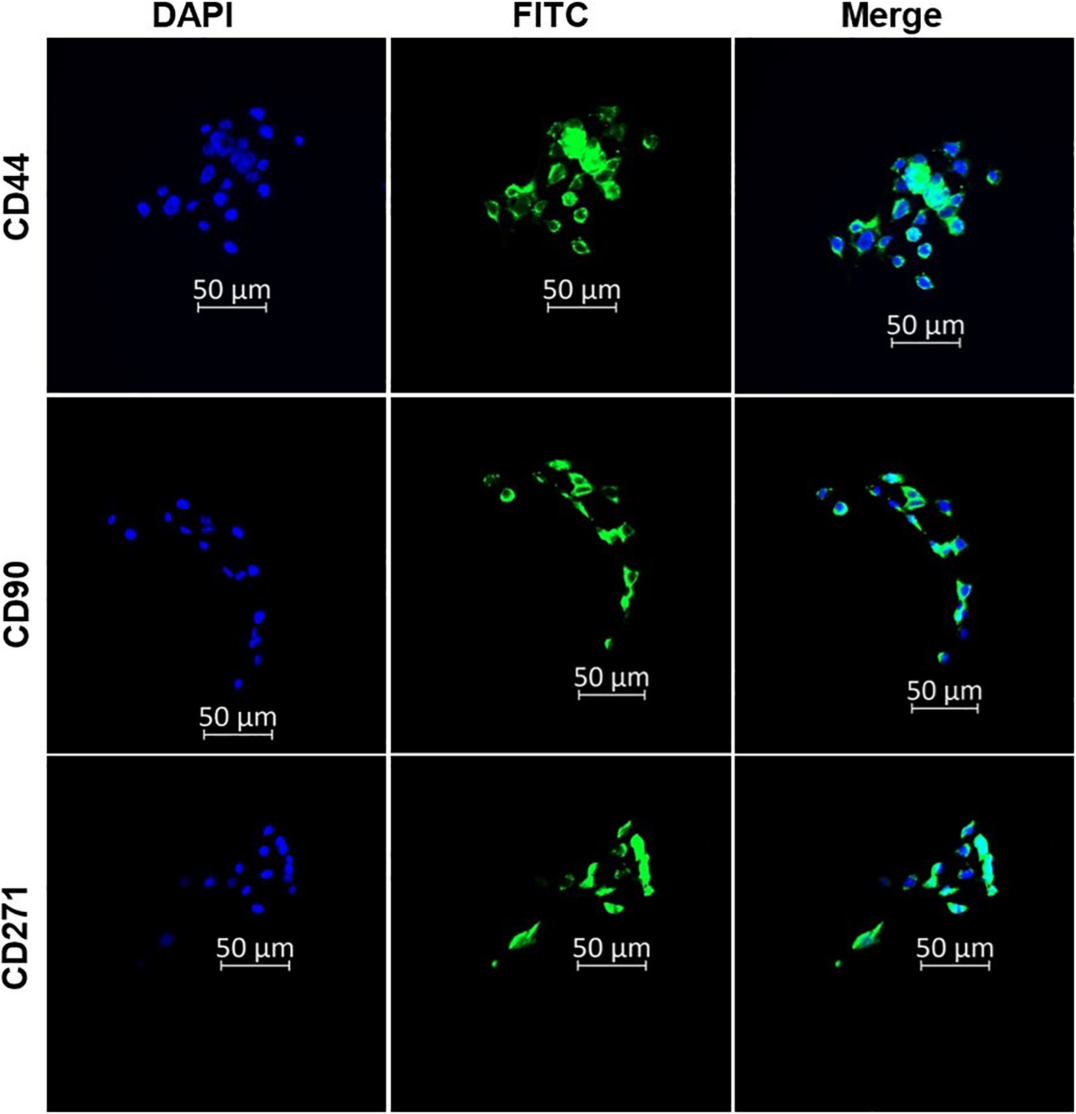
Immunofluorescence characterisation of oesophageal CSCs indicating the expression of CD44, CD90, and CD271 oesophageal CSC surface markers. The micrographs represent three independent assays (50 μm scale bar and magnification: 200x).

The surface antigen markers of the isolated CSCS were characterised using immunofluorescence microscopy. The CSCs were stained using the indirect immunofluorescence technique. The captured images showed positive expression for CD271, CD90, and CD44 surface antigen biomarkers in the CSCs (**Figure 1**). The findings confirmed that the isolated cells possess the stemness markers of oesophageal cancer cells.

### 3.2 Side population

**Figure 2:**
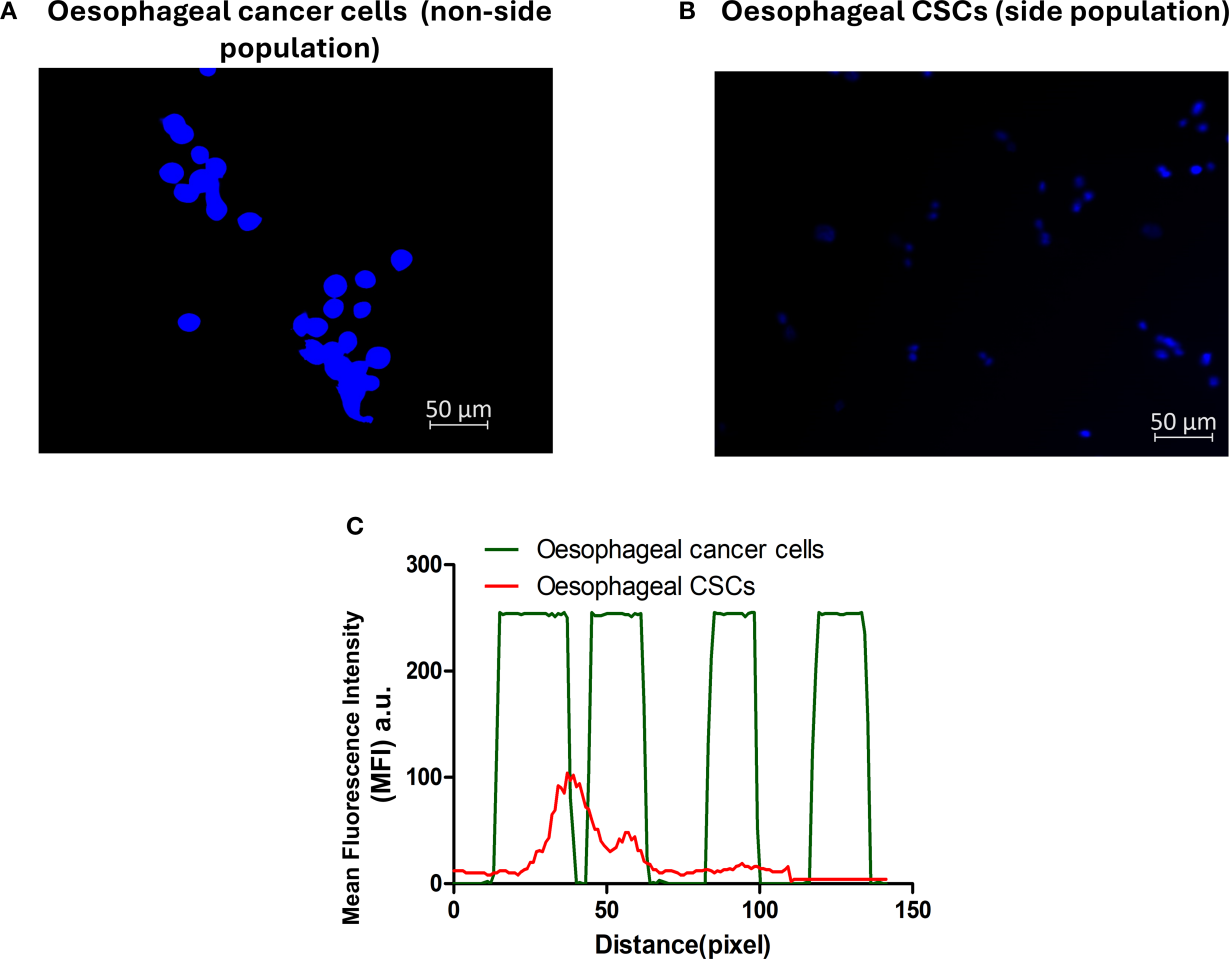
Side population characterisation using Hoechst 33342 efflux dye. Oesophageal cancer cells **(A)** and oesophageal CSCs **(B)**. **(C)** Emerged mean fluorescence intensity: oesophageal cancer cells, a non-side population, displayed a high fluorescence intensity (green line), and low fluorescence intensity was observed in the oesophageal CSCs in the side population (red line). The micrographs represent three independent assays (50 μm scale bar and magnification: 200x).

Cancer stem cells are known to efflux certain dyes, such as the Hoechst 33342 efflux dye, while cancer cells retain the dye. The isolated CSCs were distinguished from the parental tumour cells using the Hoechst 33342 efflux method. The parental oesophageal cancer cells demonstrated heavy fluorescence intensity of the blue Hoechst dye (**Figures 2A, C**), and the Isolated CSCs exhibited low fluorescence intensity of the blue Hoechst dye (**Figures 2B, C**). These results further confirm the CSCs’ attributes of the isolated cells.

### 3.3 Characterisation of the nano-immuno-conjugates

**Figure 3:**
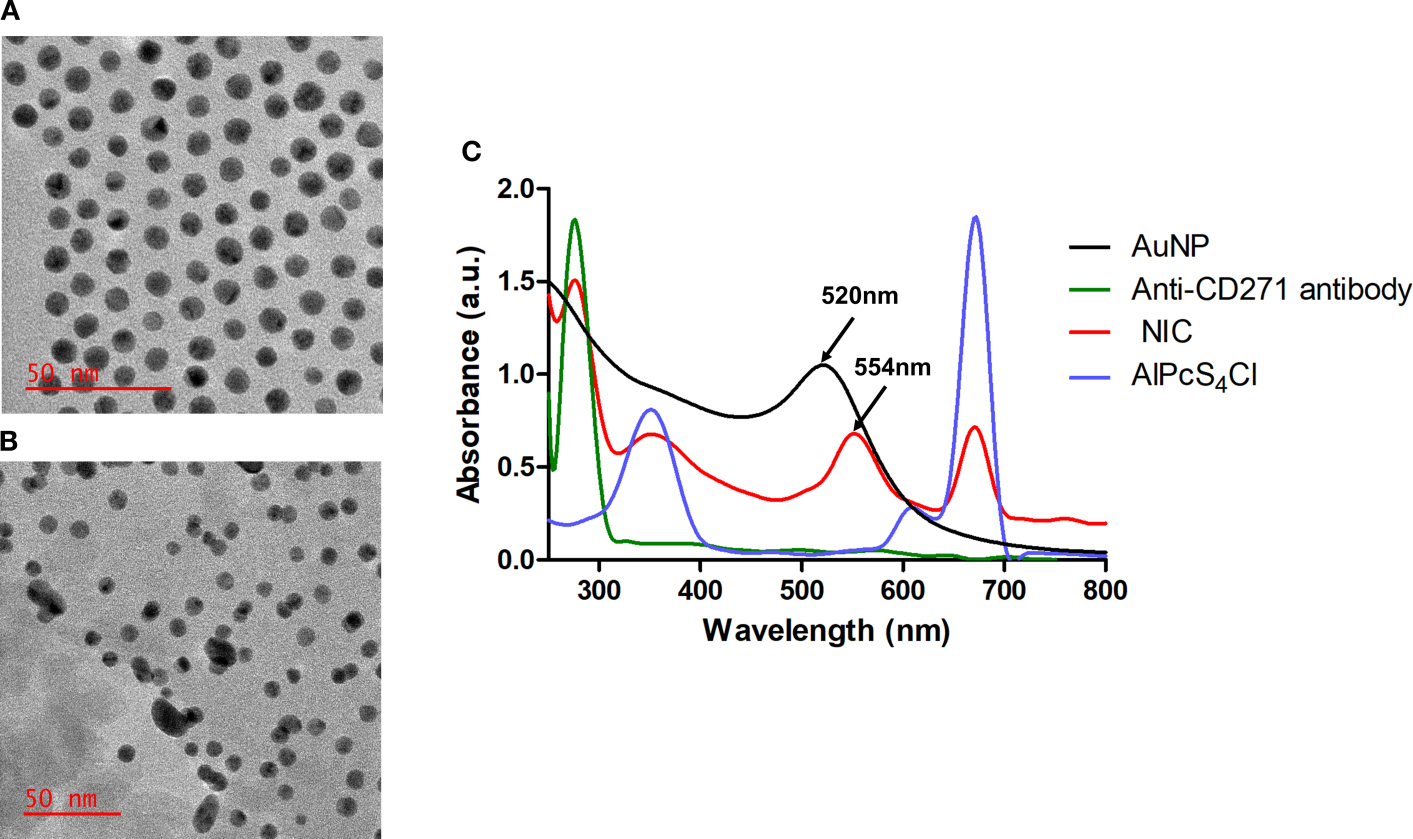
Nano-immuno-conjugate characterization. TEM analysis of AuNPs **(A)** and NIC **(B)**. **(C)** Spectra analysis of AuNPs, Anti-CD271 antibody, AlPcS_4_Cl, and NIC showing Anti-CD271 antibody at 278nm, a red shift of AuNP from 520 nm to 542 nm in the NIC and AlPcS_4_Cl at 350 nm and 672 nm.

The TEM analysis demonstrated a uniform spherical morphology of AuNPs with a mean diameter of approximately 8 nm **Figure 3A**. Meanwhile, the NIC in **Figure 3B** displayed a non-homogeneous and elongated shape with an average diameter of 11 nm when compared with the free AuNPs. This finding showed successful synthesis of the NIC. The UV-Vis spectroscopy analysis was evaluated to confirm NIC’s successful synthesis. The results in **Figure 3C** exhibited a spectrum shift in the maximum absorption band of free AuNPs from 520 nm (black curve) to 554 nm in the synthesized NIC (red curve). No spectrum shift was displayed in the absorption band of AlPcS_4_Cl at 672 nm (blue curve) and 278 nm for Anti-CD271 (green curve) before and after synthesis. However, a broad-spectrum peak ranging from 320 nm to 395 nm was observed in the synthesized NIC when compared to the free AlPcS_4_Cl, with 331 nm to 371 nm. These results further confirm the synthesis of the NIC.

### 3.4 Cellular uptake of nano-immuno-conjugates

**Figure 4:**
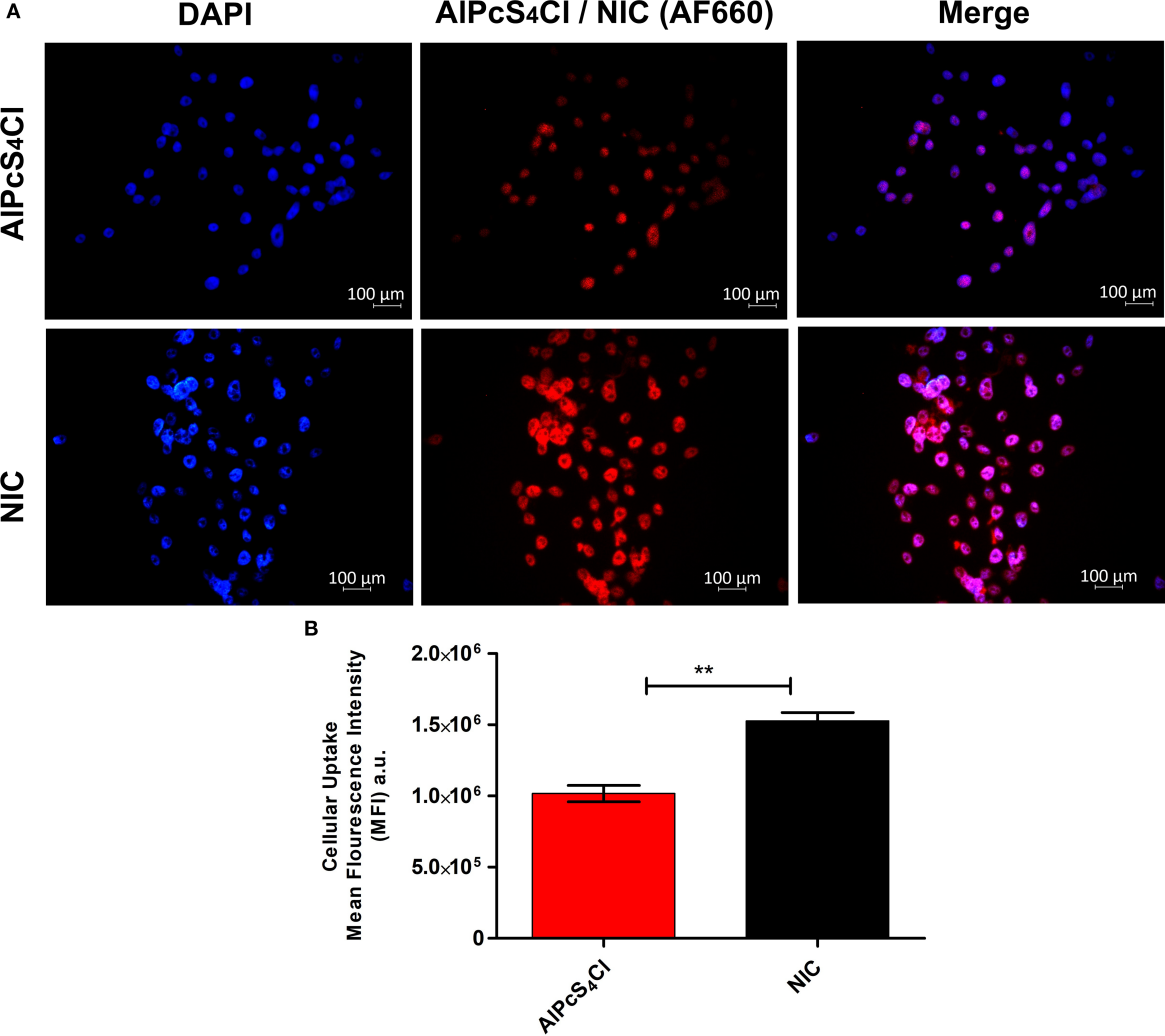
**(A)** Intracellular uptake of AlPcS_4_Cl and NIC in oesophageal CSCs after 4 hours of incubation. The AlPcS_4_Cl and NIC (red) and the nucleus of the CSCs were stained with DAPI (blue). Scale bars, 100 μm. **(B)** The MFI of three independent micrographs was determined by ZEN 3.1 software. Data represented as means ± SEM (n = 3, **p < 0.01).

Cellular uptake of NIC in oesophageal CSCs was evaluated. After treating the oesophageal CSCs with AlPcS_4_Cl and NIC for 4 hours, the CSCs treated with NIC demonstrated increased fluorescence signals compared to those with AlPcS_4_Cl (**Figure 4A**). Then, the mean fluorescence intensities (MFI) of oesophageal CSCs were measured. As depicted in **Figure 4B**, there was a 1.5-fold increase for NIC treatment compared to AlPcS_4_Cl treatment. This finding showed enhanced cellular uptake of NIC, possibly due to the AuNPs and the antibody conjugated to AlPcS_4_Cl, which improved the drug delivery and active targeting of the photosensitizer.

### 3.5 Evaluation of nano-immunoconjugates-mediated PDT and cell death mechanism on oesophageal CSCs

Cell death is a crucial activity in eukaryotic organisms that controls tissue balance and clears unhealthy and toxic cells. There are three classic categories of cell death: apoptosis, autophagy, and necrosis (50). We examined the effects of the nano-immunoconjugates (NIC) on cell viability, ROS detection, ΔΨM, efflux of cytochrome c protein, caspase 3/7 enzyme, apoptosis/necrosis, cell cycle, DNA damage response, and autophagy. The cell death mechanism induced by AlPcS_4_Cl, AlPcS_4_Cl-AuNP, and NIC-mediated PDT on oesophageal CSCs was assessed using the following concentrations: 5 µM (AlPcS_4_Cl), 5 µM:10 µM (AlPcS_4_Cl-AuNPs), and 5 µM:10 µM:5 µM (NIC (AlPcS_4_Cl: AlPcS_4_Cl-AuNPs: Anti-CD271 antibody). These concentrations were used for all experiments conducted in this study except where otherwise stated.

#### 3.5.1 Effects of nano-immunoconjugates on oesophageal CSC viability

**Figure 5:**
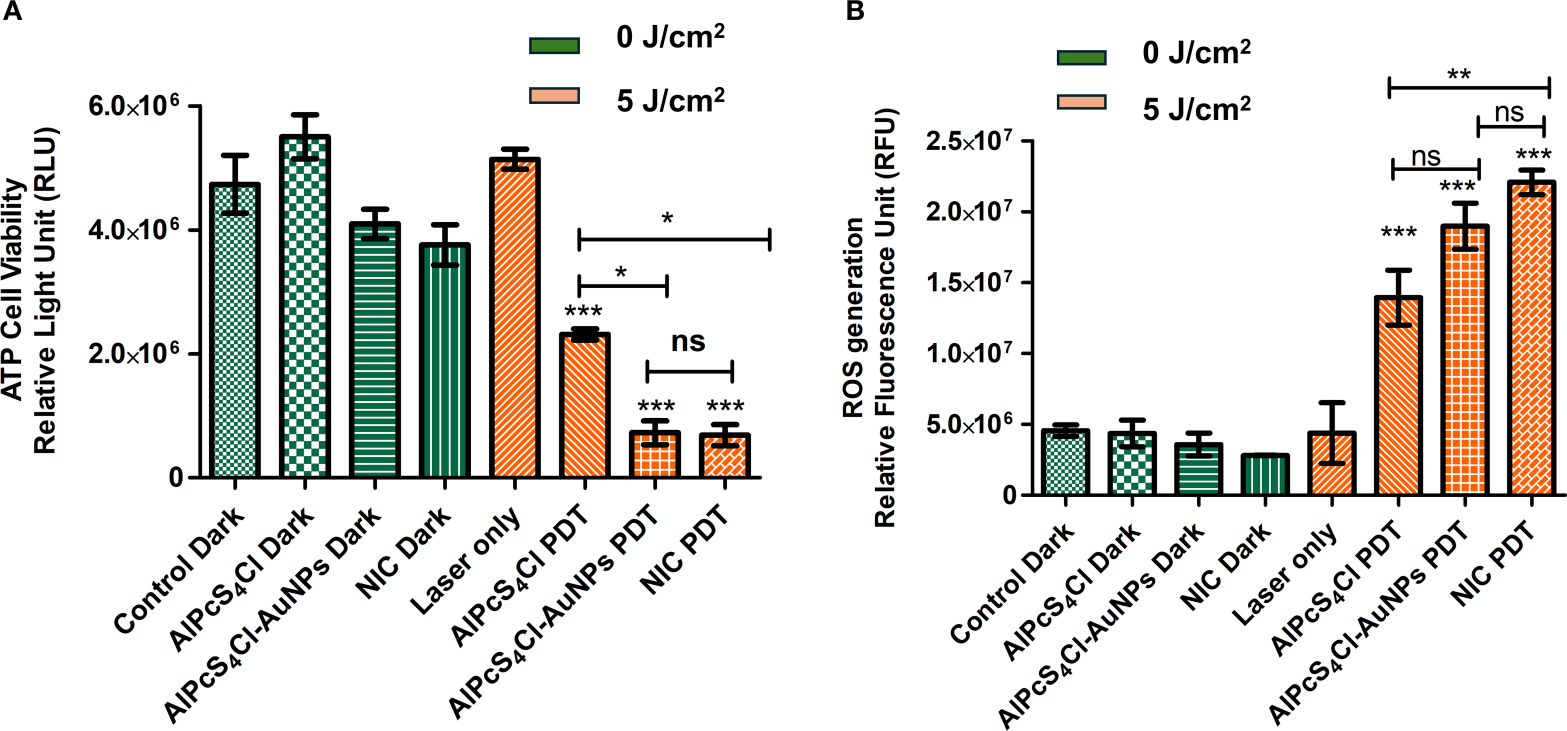
**(A)** Cell viability of oesophageal CSC exposed to AlPcS_4_Cl, AlPcS_4_Cl-AuNP, and NIC-mediated PDT. The findings showed no significant differences among the treated groups (AlPcS_4_Cl dark, AlPcS_4_Cl-AuNP dark, and NIC dark) and the control dark, at 0 J/cm^2^. While significant reduction in ATP levels and cell viability was observed in the AlPcS_4_Cl -PDT, AlPcS_4_Cl-AuNP-PDT, and NIC-PDT treated CSCs at 5 J/cm^2^ when compared to the control groups (control dark, AlPcS_4_Cl dark, AlPcS_4_Cl-AuNP dark, and NIC dark) at 0 J/cm^2^ (***p< 0.001). The AlPcS_4_Cl-AuNP-PDT and NIC-PDT treated group displayed reduced cell viability in comparison to AlPcS_4_Cl-PDT alone (*p< 0.05). No significance (ns) was shown between AlPcS4Cl-AuNPs PDT and NIC PDT. **(B)** The amount of ROS produced by AlPcS_4_Cl PDT, AlPcS_4_Cl-AuNPs PDT, and NIC PDT-treated oesophageal CSCs. The non-irradiated CSCs (green bars) and the laser-only CSCS showed no significant ROS production. The irradiated AlPcS_4_Cl PDT, AlPcS_4_Cl-AuNPs PDT, and NIC PDT on the CSCs (orange bars) showed a high production of ROS when compared to the control groups at 0 J/cm^2^ (***p< 0.001), with more ROS production in the NIC PDT group. No significance (ns) was demonstrated between AlPcS4Cl PDT and AlPcS4Cl-AuNPs PDT, and AlPcS4Cl-AuNPs PDT and NIC PDT. However significant difference was noted between AlPcS4Cl PDT and NIC PDT (**p<0.01). The results are displayed as the mean ± standard error of the mean (SEM) (n = 3).

Metabolically active and viable cells are identified by their high ATP levels and can be measured using the luminescence assay. Here, we examined the ATP levels of viable cells in the non-irradiated and irradiated groups. In **Figure 5A**, high ATP levels were observed in the non-irradiated (green bars), with no statistical difference noted in the control cells and cells receiving AlPcS_4_Cl, AlPcS_4_Cl-AuNP, and NIC at 0 J/cm^2^ (dark toxicity) when compared with the irradiated group. The results demonstrated that inactivated AlPcS_4_Cl, AlPcS_4_Cl-AuNP, and NIC do not alter the ATP levels of the cells. The irradiated groups (**Figure 5A**, orange bars) administered with AlPcS_4_Cl, AlPcS_4_Cl-AuNP, and NIC-mediated PDT at 5 J/cm^2^ displayed a substantially low level of ATP and decreased cell viability in the oesophageal CSCs (***p< 0.001) in comparison to the control cells (**Figure 5A**, green bars). The AlPcS_4_Cl-AuNP and NIC-treated cells displayed a robust reduction in cell viability compared to the free AlPcS_4_Cl-treated cells (*p< 0.05). These findings confirmed the enhanced therapeutic effects of the AlPcS_4_Cl-AuNP and NIC nano-conjugates mediated PDT.

#### 3.5.2 ROS generation induced by nano-immunoconjugates-mediated PDT on oesophageal CSCs

To evaluate the effects of AlPcS_4_Cl PDT, AlPcS_4_Cl-AuNPs PDT, and NIC PDT on oesophageal CSCs in the generation of ROS, the DCFDA/H2DCFDA cellular ROS assay was conducted. The non-irradiated groups showed no significant ROS production (**Figure 5B**, green bars). Hence, the non-irradiated cells and the laser-only cells did not generate ROS. The irradiated groups consisting of AlPcS_4_Cl PDT, AlPcS_4_Cl-AuNPs PDT, and NIC PDT treated CSCs at 5 J/cm^2^ (**Figure 5B**, orange bars) demonstrated significantly high ROS generation (***p<0.001) when compared to the non-irradiated cells. The NIC PDT displayed more ROS production among the irradiated PDT groups, followed by AlPcS_4_Cl-AuNPs PDT and the free AlPcS_4_Cl PDT. The PDT irradiated groups found that photoactivation is a crucial parameter for effective ROS production in PDT-mediated treatment.

#### 3.5.3 Effects of nano-immunoconjugates-mediated PDT on oesophageal CSCs mitochondrial membrane and efflux cytochrome c

**Figure 6:**
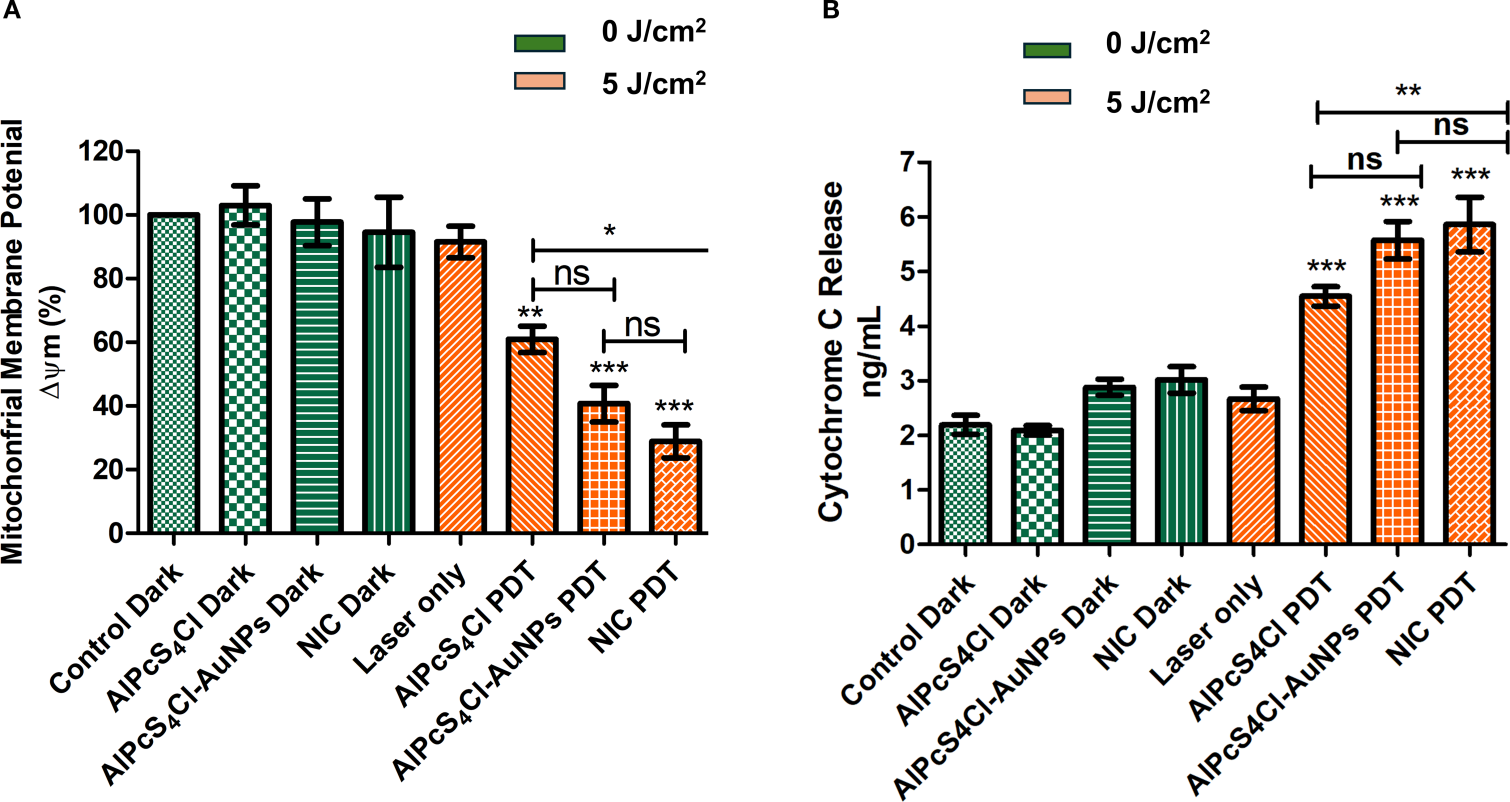
**(A)** The impact of AlPcS_4_Cl PDT, AlPcS_4_Cl-AuNPs PDT, and NIC PDT on the Δψm of oesophageal CSCs. Rhodamine-123 dye was used to examine the Δψm. High Δψm was observed among the non-irradiated control cells (green bars) and the laser-only cells, with no significance noted. The irradiated PDT-treated CSCs at 5 J/cm^2^ irradiation (orange bars), except for the laser-only cells, demonstrated significant reduction in the Δψm (**p<0.01, ***p < 0.001) in the AlPcS_4_Cl PDT, AlPcS_4_Cl-AuNPs PDT, and NIC PDT. No significance (ns) was observed between AlPcS4Cl PDT and AlPcS4Cl-AuNPs PDT, and AlPcS4Cl-AuNPs PDT and NIC PDT. While significant difference was noted between AlPcS4Cl PDT and NIC PDT (*p<0.05). The results are displayed as the mean ± SEM (n = 3). **(B)** Measurement of cytochrome c levels in AlPcS_4_Cl, AlPcS_4_Cl-AuNPs, and NIC-treated non-irradiated and irradiated oesophageal CSCs. The non-irradiated CSCs (control, AlPcS_4_Cl, and AlPcS_4_Cl-AuNPs) (green bars) and the laser-only cells showed reduced cytochrome c levels. PDT-treated cells (orange bars) exhibited statistically significantly high levels of cytochrome c (***p<0.001). No significance (ns) was observed between AlPcS4Cl PDT and AlPcS4Cl-AuNPs PDT, and AlPcS4Cl-AuNPs PDT and NIC PDT. While significant difference was noted between AlPcS4Cl PDT and NIC PDT (**p<0.01). The findings are expressed as the mean ± SEM (n = 3).

The effects of AlPcS_4_Cl, AlPcS_4_Cl-AuNPs, and NIC on the mitochondrial membrane potential (Δψm) of oesophageal CSCs were interrogated using a rhodamine-123 dye exclusion fluorimetry analysis. The percentage of Δψm was evaluated, and treatment groups were compared to the cells without treatment (**Figure 6A**). The non-irradiated and laser-only cells showed high Δψm, reflecting healthy and undamaged mitochondria. No statistical significance was noted in the non-irradiated cells (green bars) and the laser-only cells. While in the PDT-exposed cells at 5 J/cm^2^, a statistically significant reduction of Δψm (***p<0.05) was observed among the AlPcS_4_Cl PDT, AlPcS_4_Cl-AuNPs PDT, and NIC PDT-treated CSCs (orange bars) when compared to the non-irradiated control CSCs (**Figure 6A**). The findings showed that AlPcS_4_Cl PDT, AlPcS_4_Cl-AuNPs PDT, and NIC PDT-treated CSCs induced damage to the mitochondria, suggesting a possible mitochondria-dependent cell death mechanism.

The impacts of the NIC-mediated PDT in triggering the release of cytochrome c were evaluated. The findings demonstrated negligible activity of cytochrome c in the cells without irradiation, as shown in **Figure 6B** (green bars), without any statistical difference. The level of cytochrome c demonstrated in the cell receiving no irradiation suggested viable and intact mitochondria. An identical finding was exhibited in the laser-only irradiated cells displaying low cytochrome c activity. The CSCs group receiving AlPcS_4_Cl PDT, AlPcS_4_Cl-AuNPs PDT, and NIC PDT in **Figure 6B** (orange bars) exhibited significantly increased amounts of cytochrome c (***p<0.001), with the NIC PDT cells demonstrating the highest cytochrome c release. The results also showed that the nano-conjugates mediated PDT exhibited enhanced effects compared to the free drug (**p<0.01). These findings imply damage to the mitochondria; hence, the efflux of cytochrome c protein into the cytosol. This finding further affirmed the mitochondrial-dependent apoptotic mechanism triggered by the AlPcS_4_Cl PDT, AlPcS_4_Cl-AuNPs PDT, and NIC PDT on oesophageal-treated CSCs.

#### 3.5.4 Nano-immunoconjugates-mediated PDT stimulate the activation of caspase 3/7 oesophageal CSC

**Figure 7:**
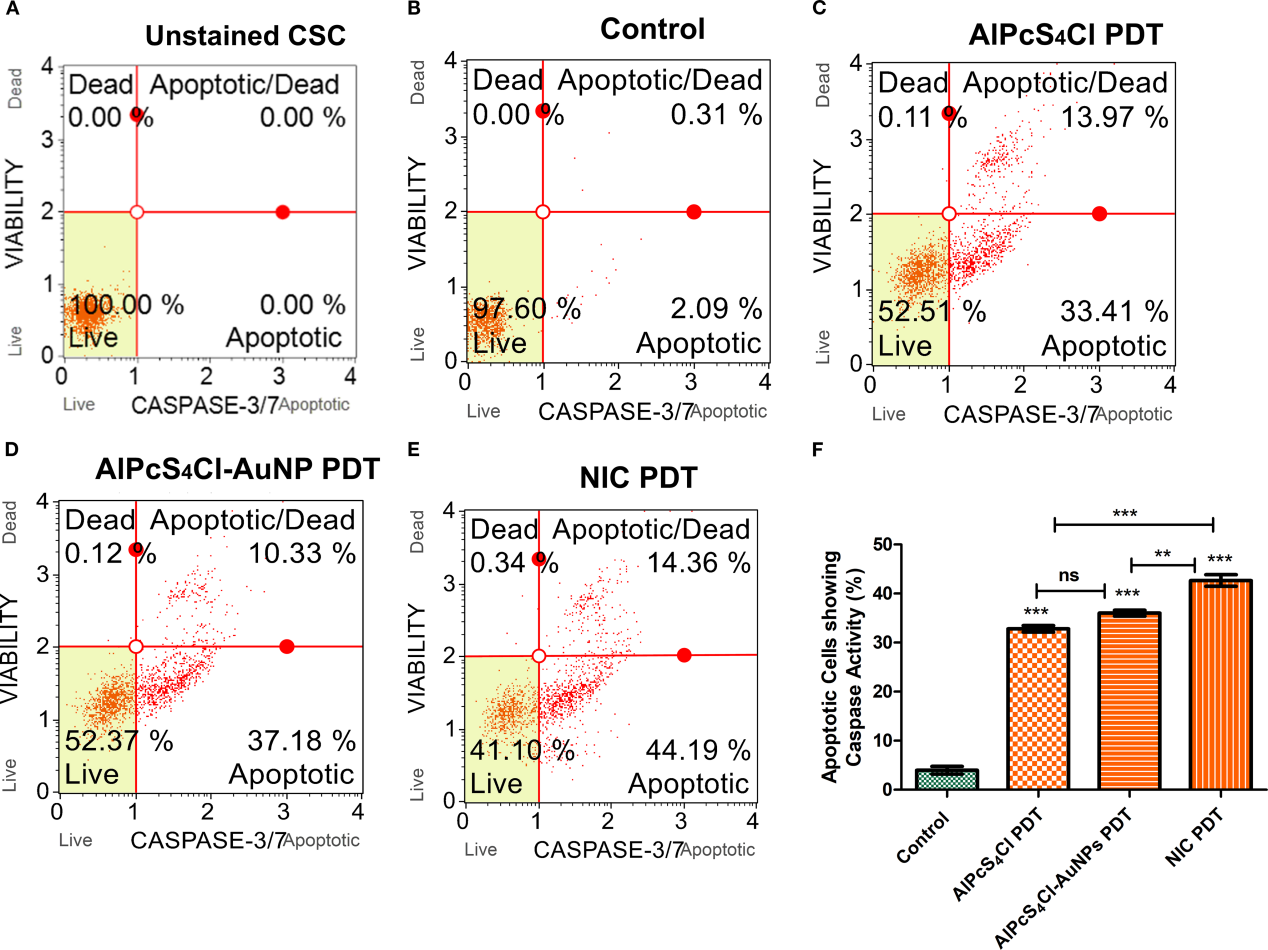
Caspase 3/7 activation and apoptotic cell evaluation of AlPcS4Cl PDT, AlPcS4Cl-AuNPs PDT, and NIC PDT on oesophageal CSCs. **(A-E)** are unstained CSC, control, AlPcS4Cl-PDT, AlPcS4Cl-AuNPs PDT and NIC PDT respectively displayed in quadrants showing 1) live cells not undergoing detectable apoptosis lower left; 2) apoptotic cells exhibiting caspase-3/7 activity lower right; 3) cells in the late stages of apoptosis or dead by necrotic or other apoptotic mechanisms upper right; and 4) cells that have died, not through the apoptotic pathway upper left. (F) Percentage measurement of three independent caspase 3/7 assays of AlPcS4Cl PDT, AlPcS4Cl-AuNPs PDT, and NIC PDT on oesophageal CSCs. The AlPcS4Cl PDT, AlPcS4Cl-AuNPs PDT, and NIC PDT-treated cells demonstrated a significant difference in caspase 3/7 activity compared to those without treatment (**p<0.01, ***p<0.001). The results are expressed as the mean ± SEM (n = 3), ns, not significant.

The activation of caspase 3/7 protease by AlPcS_4_Cl PDT, AlPcS_4_Cl-AuNPs PDT, and NIC PDT on oesophageal CSCs was investigated using the caspase-3/7 flow cytometry assay. The unstained CSCs in **Figure 7A** was used to gate various samples. The control cells in **Figure 7B** received no treatment and exhibited 97.60% live cells, 2.09% caspase 3/7 apoptotic activity, and 0.31% late apoptosis. The AlPcS_4_Cl PDT cells in **Figure 7C** demonstrated 52.51% live cells, 0.11% dead cells, 33.41% caspase 3/7 apoptotic activity, and 13.97% late apoptosis. The AlPcS_4_Cl-AuNPs PDT cells showed 52.37% live cells, 0.12% dead cells, 37.18% caspase 3/7 apoptotic activity, and 10.33% late apoptosis (**Figure 7D**). The NIC PDT cells showed 41.10% live cells, 0.34% dead cells, 44.19% caspase 3/7 apoptotic activity, and 14.36% late apoptosis (**Figure 7E**). All the PDT-treated groups displayed <1% dead cells, confirming that AlPcS_4_Cl PDT, AlPcS_4_Cl-AuNPs PDT, and NIC PDT do not induce cell death via necrosis. In addition, in **Figure 7F**, the percentage evaluation of three independent caspases 3/7 assays of AlPcS_4_Cl PDT, AlPcS_4_Cl-AuNPs PDT, and NIC PDT on oesophageal CSCs was analysed to establish the statistical variations between the control and the PDT cells. High statistical significance of caspase 3/7activity was observed in the AlPcS_4_Cl PDT, AlPcS_4_Cl-AuNPs PDT, and NIC PDT-treated oesophageal CSCs (***p<0.001) when compared to the cells receiving no treatment. The results further confirmed the apoptotic pathway induction, as demonstrated by the increased activation of caspase 3/7 in the PDT groups. When compared among the PDT group, the nano-conjugates demonstrated enhanced caspase 3/7 activity compared to the unconjugated drug.

#### 3.5.5 Evaluation of apoptosis and necrosis in oesophageal CSCS treated with nano-immunoconjugates-mediated PDT

**Figure 8:**
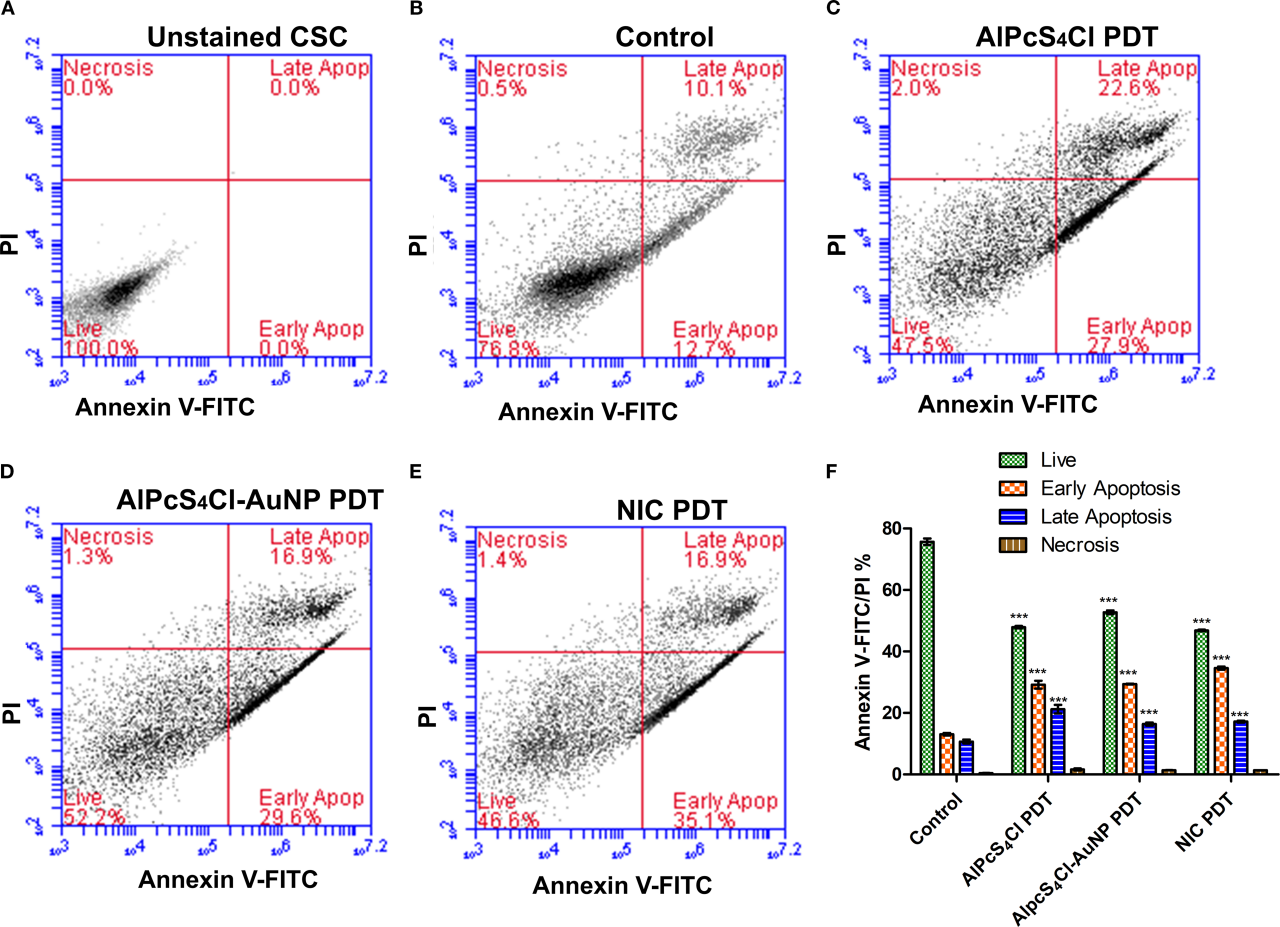
Apoptosis and necrosis cell death analysis of AlPcS_4_Cl PDT, AlPcS_4_Cl-AuNPs PDT, and NIC PDT on oesophageal CSCs. Quadrant demonstration of various stages of cell death **(A-E)** (unstained CSC, control, AlPcS_4_Cl-PDT, AlPcS_4_Cl-AuNPs-PDT, and NIC-PDT). **(F)** Percentage quantification of three independent cell death assays of AlPcS_4_Cl PDT, AlPcS_4_Cl-AuNPs PDT, and NIC PDT on oesophageal CSCs. The AlPcS_4_Cl PDT, AlPcS_4_Cl-AuNPs PDT, and NIC PDT-treated cells demonstrated highly significant differences in live cells, early apoptosis, and late apoptosis compared with untreated cells (***p<0.001). The findings are displayed as the mean ± SEM (n = 3).

Apoptotic and necrotic cell death triggered by AlPcS_4_Cl PDT, AlPcS_4_Cl-AuNPs PDT, and NIC PDT on oesophageal CSCs were investigated using the Annexin V-FITC/PI cell death analysis. The CSCs were grouped into five experiment groups: unstained cells (negative control), control with no treatment, AlPcS_4_Cl PDT, AlPcS_4_Cl-AuNPs PDT, and NIC PDT. The unstained cells (**Figure 8A**), demonstrating 100% live cells, were utilised to determine the cell population. The control cells in **Figure 8B** received no treatment and showed 76.8% live cells, 0.5% necrosis, 12.7% early apoptosis, and 10.1% late apoptosis. The AlPcS_4_Cl PDT cells in **Figure 8C** showed 47.5% live cells, 2.0% necrosis, 27.9% early, and 22.6% late apoptosis. The AlPcS_4_Cl-AuNPs PDT cells displayed 52.2% live cells, 1.3% necrosis, 29.6% early, and 16.9% late apoptosis (**Figure 8D**). The NIC PDT cells exhibited 46.6% live cells, 1.4% necrosis, 35.1% early apoptosis, and 16.9% late apoptosis (**Figure 8E**).

Furthermore, in **Figure 8F**, the percentage evaluation of three independent cell death assays of AlPcS_4_Cl PDT, AlPcS_4_Cl-AuNPs PDT, and NIC PDT on oesophageal CSCs was conducted to establish any relevant difference between the control and the PDT-treated cells. The AlPcS_4_Cl PDT, AlPcS_4_Cl-AuNPs PDT and NIC PDT treated cells demonstrated a substantially reduced number of live cells (47.39 ± 0.55%, 52.19 ± 0.67%, 46.34 ± 0.53%)(***p<0.001), significant high early apoptosis (29.12 ± 0.8, 30.32 ± 0.50, 34.58 ± 0.30)(***p<0.001), and significant increase of late apoptosis (20.92 ± 0.86, 16.07 ± 0.44, 17.14 ± 0.18) (***p<0.001) respectively in relation with the control cells. The NIC PDT-treated cells displayed a slight increase in early apoptosis when compared with the AlPcS_4_Cl PDT and AlPcS_4_Cl-AuNPs PDT group. Overall the PDT treatment groups demonstrated the induction of early apoptotic cell death.

#### 3.5.6 Nano-immunoconjugates-mediated PDT promotes G0/G1 cell cycle arrest in oesophageal CSCs

**Figure 9:**
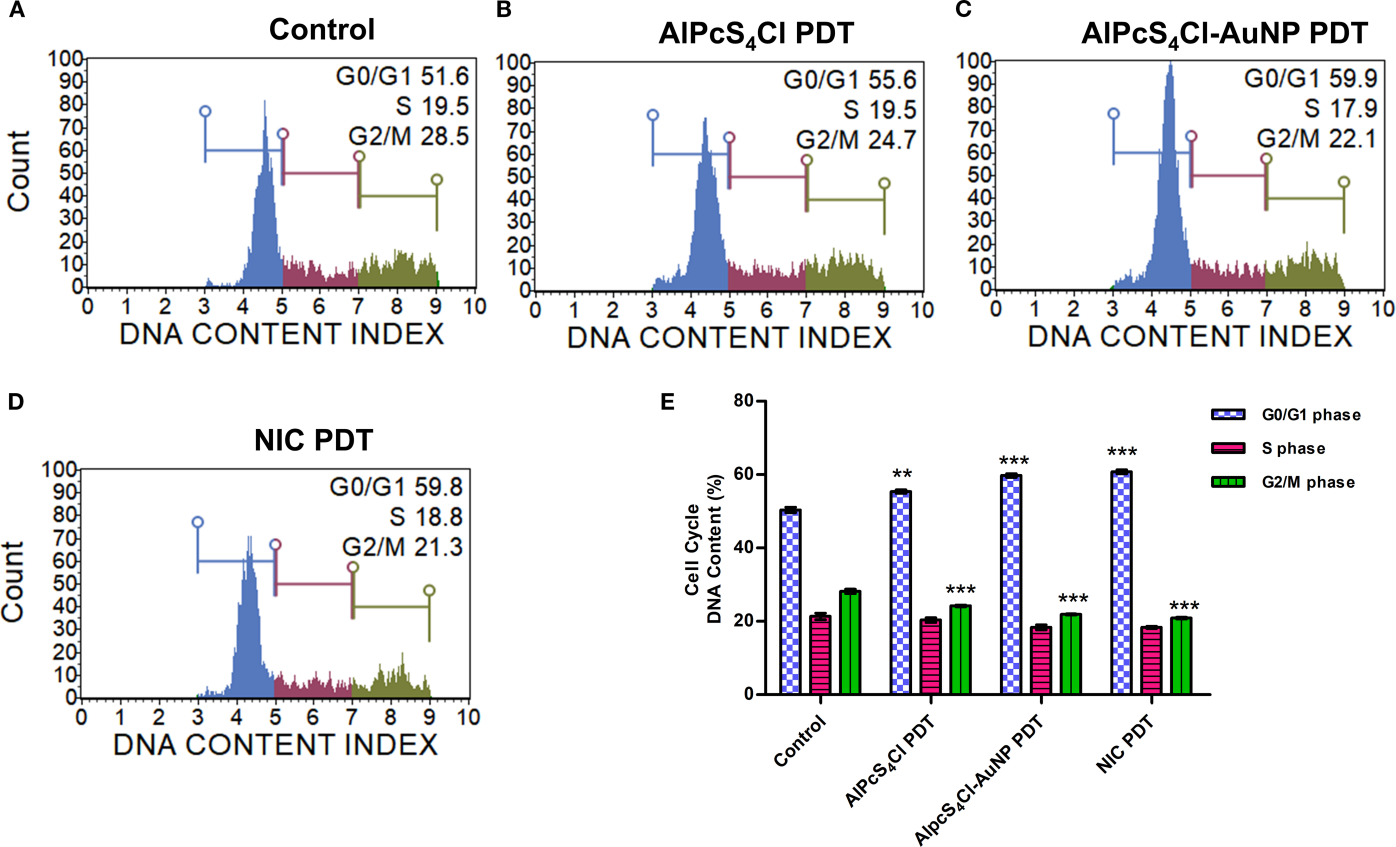
Cell cycle analysis of oesophageal CSCs. The cell distribution of the different phases of the cell cycle. **(A)** control untreated cells, **(B)** AlPcS_4_Cl-PDT, **(C)** AlPcS_4_Cl-AuNP PDT, and **(D)** NIC-PDT cells. The cell distribution at G0/G1, S, and G2/M phases of the cell cycle is indicated as blue, red, and green histograms, respectively. **(E)** Percentage distribution of DNA content of the different phases of the cell cycle with AlPcS_4_Cl, AlPcS_4_Cl-AuNP, and NIC PDT treatment groups displaying increased DNA content at the G0/G1 phase in comparison with the control, untreated cells (**p < 0.01, ***p < 0.001). The results are expressed as the mean ± SEM (n=3).

The effects of AlPcS_4_Cl, AlPcS_4_Cl-AuNP, and NIC-based PDT on the cell cycle were examined on the CSCs. (**Figures 9A–D**) displays the cell cycle distribution of oesophageal CSCs after 24 hours of PDT exposure, showing the amount of DNA content in the different cell cycle steps in the various study groups. High DNA content at the G0/G1 checkpoint was demonstrated in the AlPcS_4_Cl, AlPcS_4_Cl-AuNP, and NIC-mediated PDT cells in comparison to the control, untreated cells (**Figure 9E**, blue bars)(**p<0.01, ***p<0.001). Implying a cell cycle halt at G0/G1 phase. The nano-conjugates (AlPcS_4_Cl-AuNP and NIC-mediated PDT) groups exhibited higher and similar G0/G1 cell cycle arrest than the unconjugated AlPcS_4_Cl-PDT exposed cells. The increase in DNA contents in the G0/G1 phase shown in the PDT-exposed groups prevented the cell from entering the S phase (**Figure 9E**, red bars) and G2/M phase (**Figure 9E**, green bars), as demonstrated by the reduced DNA contents in the PDT exposure groups in comparison with the cells without treatment (***p<0.001). These results revealed that the free drug and the nano-conjugates-based PDT drive the G0/G1 cell cycle checkpoint in oesophageal CSCs. However, the nano-conjugates showed slight increase in the G0/G1 cell cycle arrest when compared with AlPcS_4_Cl-mediated PDT.

#### 3.5.7 Nano-immunoconjugate-based PDT promotes the activation of ATM and DNA double-strand breaks in oesophageal CSCs

**Figure 10:**
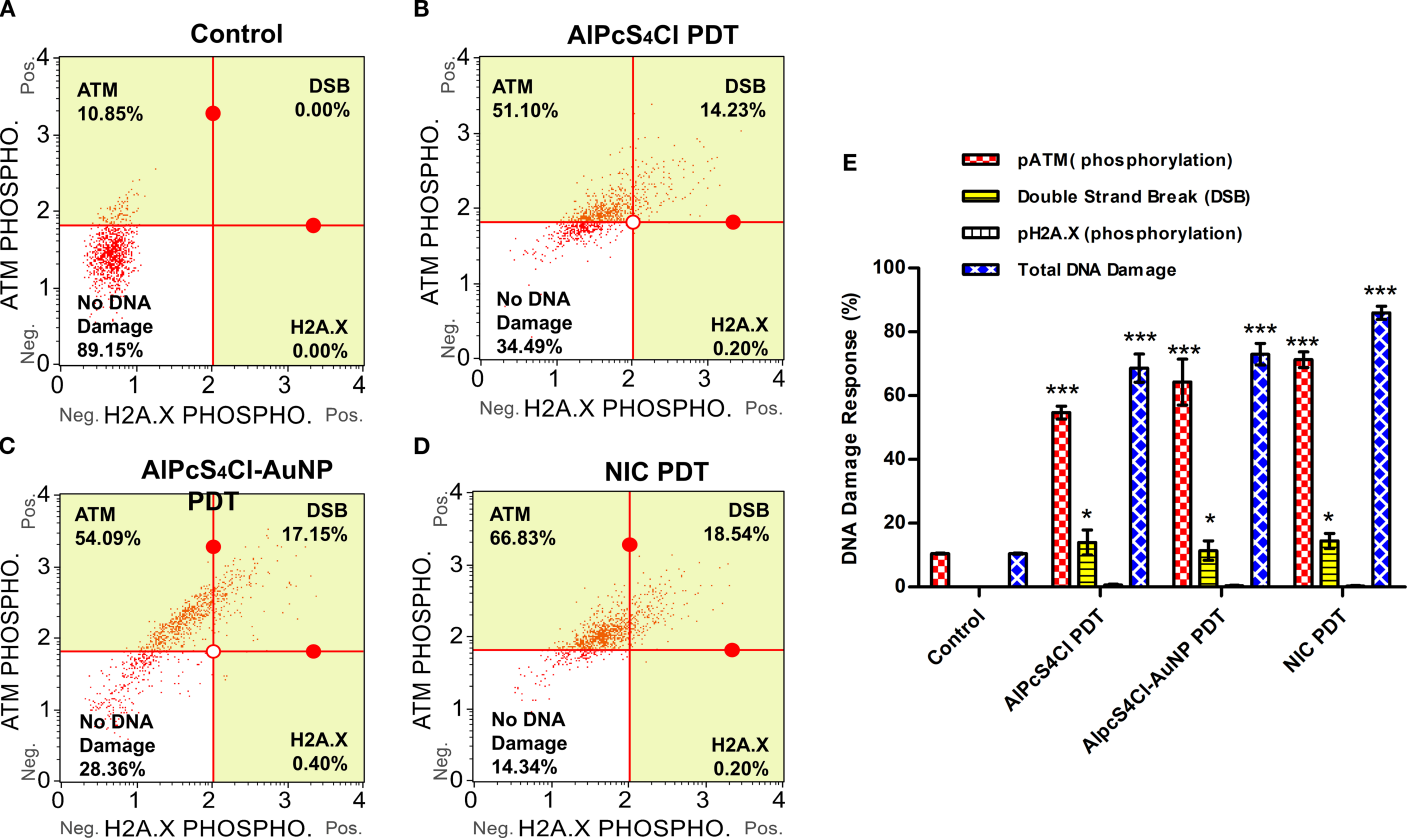
DNA damage response analysis of AlPcS_4_Cl PDT, AlPcS_4_Cl-AuNPs PDT, and NIC PDT on oesophageal CSCs. The quadrant displayed the cell expression of ATM (upper left), H2A.X (lower right), and double-strand break (DSB) (upper right). The expression of ATM, H2A.X, and DSBs in response to DNA damage in the various treatment groups: **(A)** control cells, **(B)** AlPcS_4_Cl PDT, **(C)** AlPcS_4_Cl-AuNPs PDT, and **(D)** NIC PDT. **(E)** The percentage of DNA damage response of at least three independent assays is represented in bar charts. The pATM-positive cell population (red bars) demonstrated high expression in AlPcS_4_Cl PDT, AlPcS_4_Cl-AuNPs PDT, and NIC PDT-exposed CSCs in contrast to the cells with no treatment control (***p<0.001). The DSB (yellow bars, activated pATM/pH2A.X-positive cells) shows expression in the CSCs exposed to AlPcS_4_Cl PDT, AlPcS_4_Cl-AuNPs PDT, and NIC PDT than the control cells (*p<0.05). The pH2A.X-positive cells showed expression of < 1% with no significance. The total DNA damage (blue bars) displayed substantial damage in all the treated cells in comparison to the control CSCs (***p<0.001). The findings are denoted as ± SEM (n = 3).

The DNA damage response on oesophageal CSCs treated with AlPcS_4_Cl PDT, AlPcS_4_Cl-AuNPs PDT, and NIC PDT was examined using the Muse^®^ Multi-Colour DNA Damage assay after 24-hour PDT application. This assay simultaneously identifies the activation of ATM, H2A.X, and recognition of double-strand breaks (DSBs) through co-expression of ATM/H2A.X in DNA damage response to therapeutic agents using anti-phospho-histone H2A.X and anti-phospho-ATM antibodies. (**Figures 10A–D**) shows the DNA damage response of oesophageal CSCs treated with AlPcS_4_Cl PDT, AlPcS_4_Cl-AuNPs PDT, and NIC PDT, displaying the different response mechanisms. The treatment of oesophageal CSCs with AlPcS_4_Cl-PDT, AlPcS_4_Cl-AuNPs-PDT, and NIC-PDT significantly induced DNA damage through increased ATM phosphorylation in contrast with the untreated cells (**p<0.01, ***p<0.001) as depicted in **Figure 10E** (red bars). In addition, increased DNA double-strand breaks (DSBs) were observed in the AlPcS_4_Cl PDT, AlPcS_4_Cl-AuNPs PDT, and NIC PDT-treated oesophageal CSCs, showing statistically significant DSBs in the PDT-exposed cells compared to the control cells receiving no treatment (*p<0.05) (**Figure 10E**, yellow bars).

Meanwhile, a low level of phosphorylated H2A.X was demonstrated in the PDT-treated CSCs, and no expression was seen in the control cells (**Figure 10E**). Furthermore, the result in **Figure 10E** (blue bars) showed the total percentage of DNA damage induced by AlPcS_4_Cl-PDT, AlPcS_4_Cl-AuNPs-PDT, and NIC-PDT on oesophageal CSCs, NIC PDT demonstrating the highest induction, followed by AlPcS_4_Cl-AuNPs PDT and lastly AlPcS_4_Cl-PDT when compared with the control untreated CSCs (***p<0.001). The report revealed that AlPcS_4_Cl-PDT, AlPcS_4_Cl-AuNPs-PDT, and NIC-PDT exposed to oesophageal CSCs remarkably induced DNA damage response via the ATM and double-strand break response mechanism.

#### 3.8.8 Nano-immunoconjugate-based PDT induces autophagy in oesophageal CSCs

**Figure 11:**
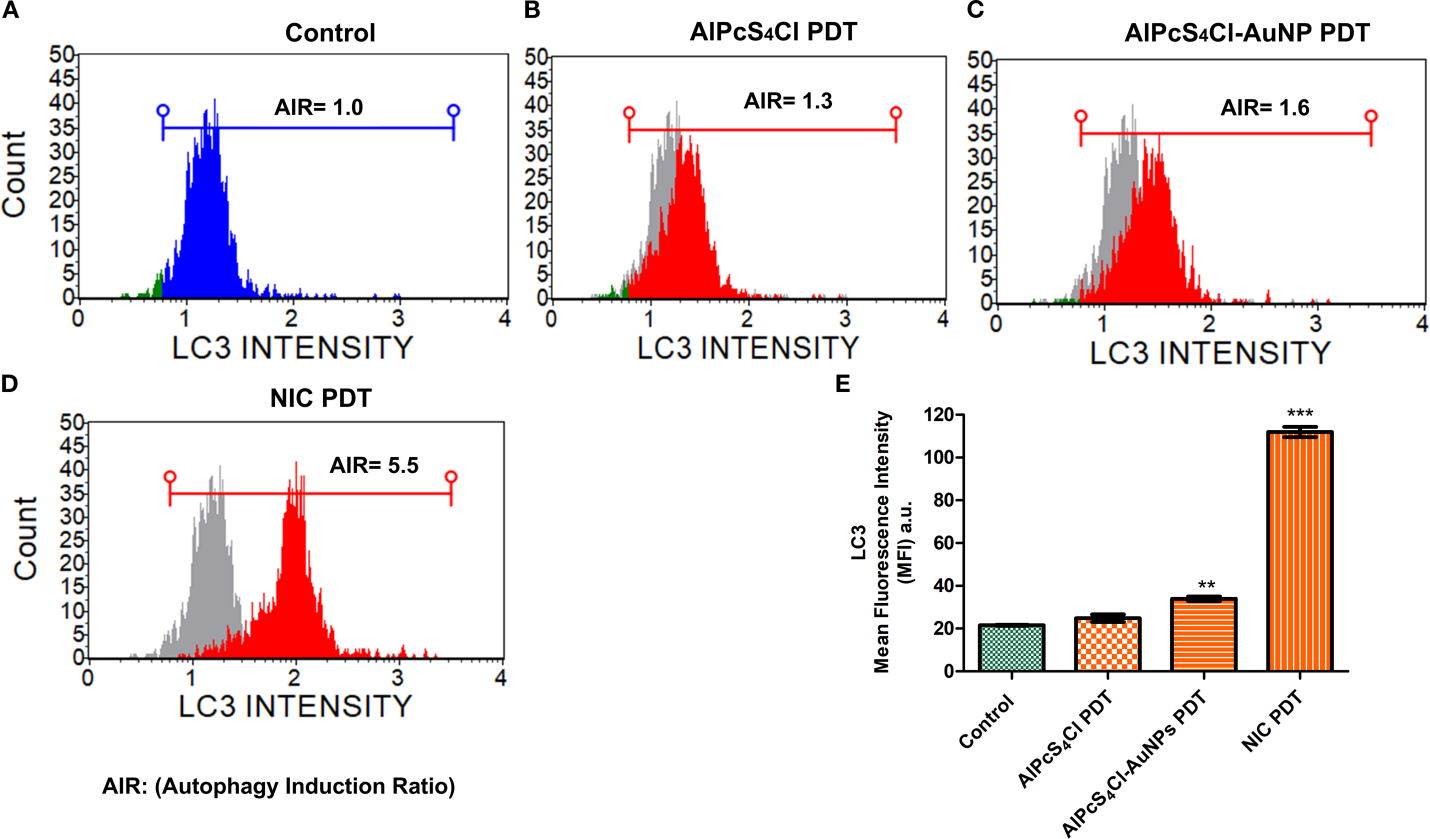
Autophagy cell death analysis. **(A)** Control without treatment with an AIR of 1.0 indicates no autophagy induction. **(B)** AlPcS_4_Cl PDT on oesophageal CSCs showing AIR of 1.3, indicating slight induction; however, it is insignificant. **(C)** AlPcS_4_Cl-AuNPs PDT treated oesophageal CSCs displaying a statistically significant 1.6 AIR (**p<0.01). **(D)** NIC PDT treated oesophageal CSCs showing a robust AIR 5.5 (***p<0.001). The samples overlapped with the control (grey peak). **(E)** Percentage measurement of three independent Autophagy LC3 antibody-based detection assays of AlPcS_4_Cl PDT, AlPcS_4_Cl-AuNPs PDT, and NIC PDT on oesophageal CSCs. The NIC PDT-treated cells demonstrated a highly significant difference in AIR compared to the AlPcS_4_Cl-PDT, AlPcS_4_Cl-AuNPs PDT, and control cells (***p<0.001). The findings are expressed as ± SEM (n = 3).

Here, autophagy cell death was analysed using the Autophagy LC3 antibody assay 24 hours post-PDT. The mean autophagy intensity values were obtained by measuring the LC3 protein intensity in the control and the treatment groups (AlPcS_4_Cl PDT, AlPcS_4_Cl-AuNPs PDT, and NIC PDT) of the oesophageal CSCs. The autophagy induction ratio (AIR) was deduced by dividing the mean autophagy signal of the treatment cells by the mean autophagy signal of the untreated cells. The control untreated in **Figure 11A** had AIR of 1.0 and was used as the AIR baseline. An AIR ≥1 indicates an autophagy response, and 1 ≤ non-response. The AIR results demonstrated that the CSCs treated with AlPcS_4_Cl PDT at 5 J/cm^2^ displayed an AIR of 1.3 when matched with the control, and no statistical significance was demonstrated (**Figures 11B, E**). The AlPcS_4_Cl-AuNPs PDT-treated CSCs showed a slight AIR (1.6) when paired with the control (**Figures 11C, E**); it is not statistically significant. While the NIC PDT-treated CSCs demonstrated a high statistically significant AIR (5.5) in contrast to the untreated control (***p<0.001) (**Figures 11D, E**). The finding showed that NIC PDT significantly triggered autophagy cell death compared to the treated PDT groups.

## 4 Discussion

This study examined the impacts of nano-immunoconjugates (NIC)-based PDT and possible cell death mechanisms on oesophageal CSCs. The NIC is a multi-component formulation consisting of AlPcS_4_Cl, AuNPs, and anti-CD271 antibody (AlPcS_4_Cl-AuNPs-anti-CD271 antibody). The oesophageal CSCs were enriched with CD44, CD90, and CD271 stemness surface antigens (biomarkers). These biomarkers used in this study are known to drive cancer relapse, tumour proliferation, treatment failure, and maintain the stemness features of oesophageal cancer (10, 47, 51). Due to the above features, CSCs expressing CD44, CD90, and CD271 stemness surface antigens were utilised as treatment populations in this study. The oesophageal CSCs were effectively isolated using the magnetic cell sorting approach and characterised by immunofluorescent protein detection and the Hoechst 33342 efflux method. Currently, nanoparticle-mediated PDT using different types of nanoparticles has been reported on various cancer types, as well as in oesophageal cancer (3, 38, 52, 53). The use of gold nanoparticles in PDT has drawn much attention as an effective delivery system for transporting photosensitiser agents to cancer cells owing to their favourable biocompatibility, chemical inertness, and strong optical properties (38, 53). The application of biomolecule-based phthalocyanine-AuNPs for enhanced PDT on various cancer types has been well-studied (38, 40, 41, 45). However, the application of gold nano-immunoconjugate-mediated PDT and the cell death mechanism on isolated oesophageal CSCs is limited. Therefore, a newly synthesized and characterized NIC was employed to evaluate the anti-tumour effects and cell death pathways activated by NIC-mediated PDT on oesophageal CSCs. The findings from the characterisation of NIC showed successful synthesis of the NIC. The findings from the TEM analysis showed that NIC were about 11 nm in diameter, and the UV-Vis spectroscopy results of NIC showed spectral shifts in the AuNPs and the AlPcS4Cl absorption bands. Findings from cellular uptake of NIC and AlPcS_4_Cl in the oesophageal CSCs showed enhanced cellular internalization in the cells receiving NIC as compared to the AlPcS_4_Cl, suggesting the integration of the AuNP and antibody facilitated the delivery of the photosensitizer into the cancer cells.

The PDT effects and cell death mechanisms of the NIC on isolated oesophageal CSCs were investigated via ATP cell viability, ROS production, mitochondrial membrane potential, leakage of cytochrome c protein, caspase 3/7 activation, annexin FITC/PI cell death mechanism, cell cycle analysis, DNA damage response pathway, and autophagy cell death detection. The effects of NIC-based PDT were compared to the control and the unconjugated AlPcS_4_Cl. Findings from cell viability evaluation showed that the cells with no treatment and the cells exposed to AlPcS_4_Cl, AlPcS_4_Cl-AuNP, and NIC treatment at 0 J/cm^2^ (no irradiation) displayed metabolically active and healthy cells with increased amount of ATP, demonstrating the non-toxicity effects of AlPcS_4_Cl, AlPcS_4_Cl-AuNP and NIC in the absence of light activation. Whereas AlPcS_4_Cl, AlPcS_4_Cl-AuNP, and NIC exposed to 5 J/cm^2^ irradiation exhibited significantly less metabolically active, viable cells and reduced amount of ATP, indicative of a cell death event. When compared among the PDT treatment groups, the nano-conjugated drugs (AlPcS_4_Cl-AuNP and NIC) triggered much more cytotoxicity effects than the free AlPcS_4_Cl. The increased effect in the nano-conjugates is possible due to the improved photosensitiser load and increased cellular uptake by the AuNPs. These findings agree with those documented by Chizenga and Abrahamse (40), Crous and Abrahamse (41), and Obaid and colleagues (54).

In physiological states, the production of cellular ATP is the primary function of the mitochondria. In contrast, in pathological conditions, the mitochondria modulate the generation of oxygen radicals and ROS, inhibiting proper generation of ATP, blocking cell proliferation, promoting the release of pro-apoptotic proteins, and cell death. Increased cellular ROS generation is linked to oxidative cellular damage (55, 56). This study showed that all the experimental irradiated drugs (AlPcS_4_Cl, AlPcS_4_Cl-AuNPs, and NIC-mediated PDT) triggered ROS generation in oesophageal CSCs compared to the control and non-irradiated cells. The NIC-mediated PDT-treated cells displayed more ROS production when compared to the AlPcS_4_Cl-AuNPs-mediated PDT and the free AlPcS_4_Cl-mediated PDT. These findings demonstrated the enhanced therapeutic efficiency of nano-conjugates and active targeting approaches in improving PDT treatment. This report aligned with a previous study showing the increased production of ROS by AuNPs-RNase A in colorectal cancer cells (57). Similarly, another study by Simelane and colleagues exhibited high ROS production when colon cancer cells were treated with aluminium phthalocyanine tetra sodium 2-mercaptoacetate coupled with pegylated copper–gold bimetallic nanoparticles based PDT on colon carcinoma *in vitro* (58). However, Obaid and colleagues found no significant ROS generation in the gold nanoparticles conjugated with antibody/lectin and the free photosensitiser (54).

A high level of cellular ROS has been found to disrupt the potential of the mitochondrial membrane, resulting in the efflux of several proteins from the mitochondria. This study evaluated the integrity of the mitochondrial membrane potential (Δψm) of oesophageal CSCs treated with AlPcS_4_Cl, AlPcS_4_Cl-AuNPs, and NIC-mediated PDT. The results demonstrated that AlPcS_4_Cl, AlPcS_4_Cl-AuNPs, and NIC-mediated PDT significantly distorted the ΔΨM of the oesophageal CSCs compared to the control and non-irradiated CSCs. The nano-conjugates, AlPcS_4_Cl-AuNPs and NIC-mediated PDT CSCs, displayed gross Δψm impairment compared to the free AlPcS_4_Cl-mediated PDT. These results agree with the increase in cellular ROS production observed in this study. This suggests that the nano-conjugates promote oxidative cellular damage and enhance the therapeutic activity of PDT in oesophageal CSCs. Previous studies have shown that damage to the mitochondrial membrane predisposes the cells to be more responsive to treatment (59, 60). Impaired ΔΨM is a vital process in the mitochondrial apoptotic cell death pathway.

The mitochondria house various proteins, including cytochrome c. Cytochrome c plays a vital function in ATP production and oxidative phosphorylation. Injury to the mitochondria results in the leakage of the cytochrome c protein, which stimulates the action of several proteins in the apoptotic signalling pathway. To confirm the impairment of the mitochondrial membrane, we examine the leakage of cytochrome c protein from the mitochondria. Damage to the mitochondrial membrane facilitates the leakage of mitochondrial proteins, such as cytochrome c, which then collaborate with several proteins in the cytosol to activate the caspases and, in turn, apoptosis. The cytoplasmic cytochrome c levels of oesophageal CSCs treated with AlPcS_4_Cl, AlPcS_4_Cl-AuNPs, and NIC-mediated PDT were investigated. Increased cytochrome c levels were observed in the oesophageal CSCs exposed to AlPcS_4_Cl, AlPcS_4_Cl-AuNPs, and NIC-mediated PDT compared to the control non-irradiated oesophageal CSCs. The findings confirmed that AlPcS_4_Cl, AlPcS_4_Cl-AuNPs, and NIC-based PDT remarkably altered the ΔΨM of the oesophageal CSCs. Hence, this suggests that the AlPcS_4_Cl, AlPcS_4_Cl-AuNPs, and NIC-based PDT induced cell death mechanism through the mitochondria-apoptosis signalling cascade in oesophageal CSCs. A comparable outcome was demonstrated in preclinical models where breast cancer cells were subjected to zinc phthalocyanine-AuNPs-based PDT (61) and zinc phthalocyanine-based PDT in melanoma cells (62). Furthermore, similar findings were demonstrated to release cytochrome c protein following mitochondrial membrane damage in various tumour cells treated with different nanomaterials (63).

The leakage of cytochrome c protein from the damaged mitochondria is vital in the upstream signalling of apoptosis and caspase-dependent cell death. In this study, caspase 3/7 activity was examined using the caspase-3/7 activation assay. It was seen that AlPcS_4_Cl, AlPcS_4_Cl-AuNPs, and NIC-mediated PDT on oesophageal CSCs demonstrated a significantly high activity of caspase 3/7 protease in contrast to the control non-irradiated, untreated CSCs. This result aligned with the study conducted by Dam and co-workers, where Aptamer-loaded gold nanostars treated cervical cancer cells, demonstrated increased caspase 3/7 activity and induction of apoptosis (64). Increased caspase activity was also shown when breast cancer was treated with zinc phthalocyanine-AuNPs-mediated PDT (61).

Moreover, to identify the exact cell death pathways mediated by AlPcS_4_Cl, AlPcS_4_Cl-AuNPs, and NIC-mediated PDT on oesophageal CSCs, we examined the annexin V-FITC/PI cell death analysis. This analysis was conducted to establish which cell death mechanism is involved in the cell damage observed. The results from the Annexin V-FITC/PI cell death analysis showed that AlPcS_4_Cl, AlPcS_4_Cl-AuNPs, and NIC-mediated PDT on oesophageal CSCs displayed substantial apoptotic cell death when compared to the control, untreated CSCs. In addition, the reports from the AlPcS_4_Cl, AlPcS_4_Cl-AuNPs, and NIC-mediated PDT on oesophageal CSCs exhibited some necrotic cell death mechanism compared to the control cells; however, these findings were minute compared to the apoptotic cell death mechanism. The apoptosis and necrosis reports showed that apoptosis is the primary cell death pathway induced by AlPcS_4_Cl, AlPcS_4_Cl-AuNPs, and NIC-mediated PDT on oesophageal CSCs. This finding agrees with the results from ROS, mitochondrial membrane potential, cytochrome c leakage, and caspase 3/7 activation, suggesting the activation of the intrinsic apoptotic cell death mechanism. This is in concordance with past findings where apoptosis was revealed to be the preferred cell death pathway induced by AlPcS_4_Cl-mediated PDT on oesophageal cancer (33) and by AuNPs-antibody-based PDT on lung CSCs (41). Another study observed apoptosis as the primary cell death mechanism triggered by zinc-phthalocyanine-gold dendrimeric nanoparticles-based PDT on breast cancer cells (61). Furthermore, pegylated AuNPs conjugated with RNase A were shown to induce apoptosis in colorectal tumour cells (57). However, these findings do not exclude the likelihood that stimulation of extrinsic apoptotic pathways may be implicated in AlPcS_4_Cl, AlPcS_4_Cl-AuNPs, and NIC-mediated PDT-triggered apoptotic cell death, as we did not investigate the extrinsic apoptotic proteins.

Furthermore, we examine the impact of AlPcS_4_Cl, AlPcS_4_Cl-AuNPs, and NIC-based PDT on the cell cycle distribution of oesophageal CSCs. Cell cycle dysregulation is among the key features of cancer; hence, targeting proteins involved in cell cycle phases may provide an effective measure to mitigate the excessive proliferation of cancer cells. Our investigation showed that AlPcS_4_Cl, AlPcS_4_Cl-AuNPs, and NIC-mediated PDT exhibited high DNA content in the G0/G1 phase. The findings highlight the remarkable effects of AlPcS_4_Cl, AlPcS_4_Cl-AuNPs, and NIC-mediated PDT in inhibiting cell growth by eliciting increased DNA content in the G0/G1 phase of oesophageal CSCs, which agrees with a past report (65). However, Zhao et al. documented high DNA content in S-phase cell cycle in bladder tumour cells exposed to polydopamine-decorated branched Au–Ag nanoparticles (66). The display increased DNA content at the G0/G1 phase observed in the AlPcS_4_Cl, AlPcS_4_Cl-AuNPs, and NIC-mediated PDT-treated oesophageal CSCs is suggested to be due to the activation of the DNA damage repair mechanism preceding DNA damage in DNA damage response (DDR) pathways.

The DDR performs a crucial function in maintaining the genomic stability of cells. Anticancer drugs that induce DNA damage evoke several signalling cascades that dictate the outcome of damaged cells. Depending on the degree of damage and the metabolic status of the cell, several signalling pathways are activated to promote damage tolerance, stop the cell cycle, repair damaged DNA, or induce cell death. Among the diverse forms of DNA damage that can be initiated by DNA-damaging substances, DNA double-strand breaks (DSBs) are found to be the most frequently observed class of DNA damage triggered by anti-tumour drugs (67, 68). The DSBs are often established by the upregulation of certain proteins, such as ataxia telangiectasia mutated (ATM). The findings from this study showed that oesophageal CSCs treated with AlPcS_4_Cl, AlPcS_4_Cl-AuNPs, and NIC-mediated PDT triggered the upregulation of ATM and DSBs, key events involved in the DNA damage response pathways. This report agrees with past findings where cells exposed to ionising radiation (69, 70) and PDT on oesophageal cancer cells (33) showed upregulation of the ATM pathway, which was remarkably linked with DSBs.

**Figure 12:**
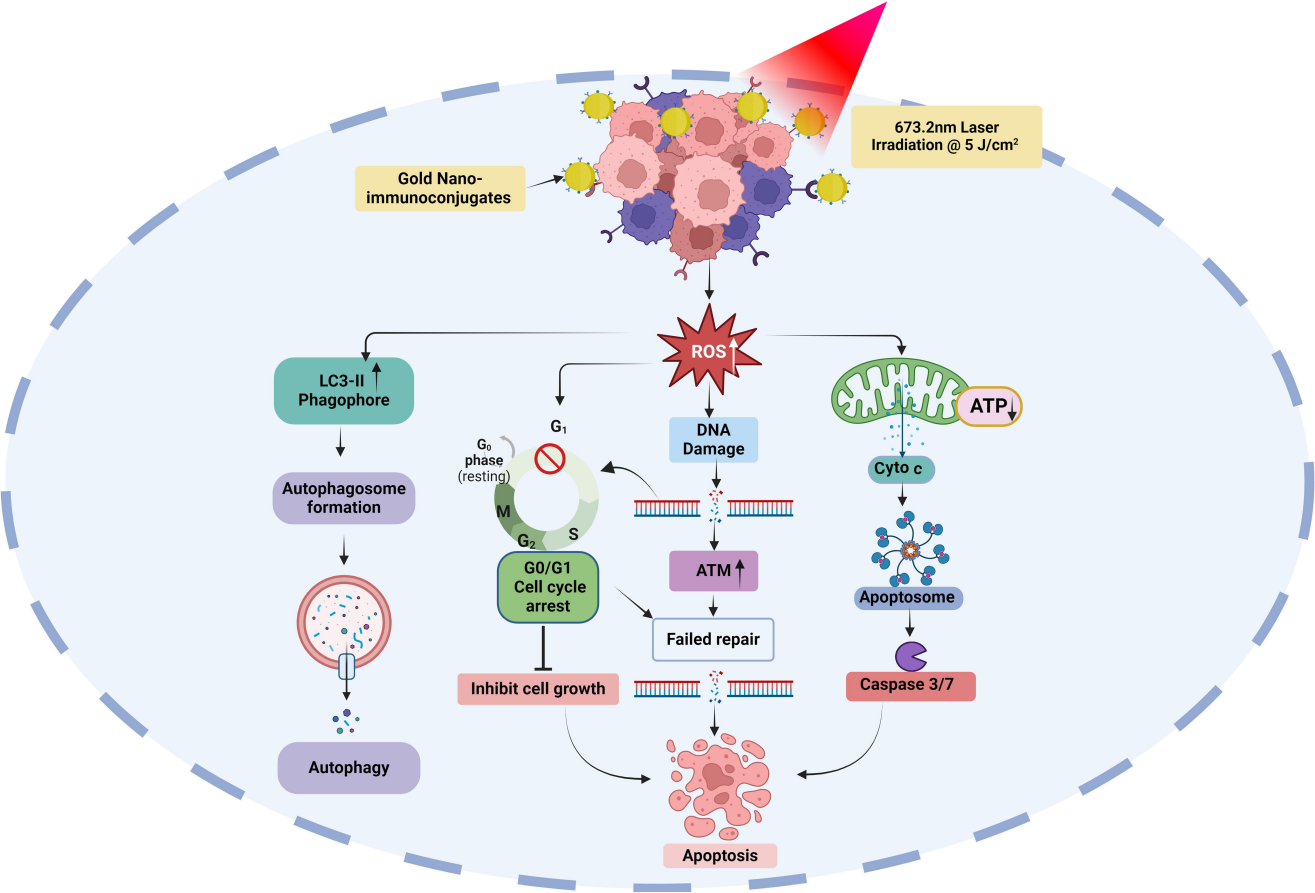
Possible molecular and cell death mechanisms of gold nano-immunoconjugate-based PDT for oesophageal CSCs. ROS, reactive oxygen species; ATP, Adenosine triphosphate; Cyto c, cytochrome c; LC3-II, microtubule-associated protein-II light chain 3; ATM, ataxia-telangiectasia mutated.

Finally, we evaluated the effects of AlPcS_4_Cl, AlPcS_4_Cl-AuNPs, and NIC-mediated PDT on autophagic cell death in oesophageal CSC. Autophagy is a self-degradation system that plays a vital role in cellular equilibrium by removing and reprocessing defective cellular structures. It is an auto-degradation process that reacts to stimuli such as DNA damage, metabolic imbalance, nutrient depletion, hypoxia, and anti-tumour agents (71, 72). This process can either promote cell survival or cell death. Usually, autophagy functions as a cell survival mechanism; however, dysregulation of autophagy mechanisms or increased autophagic flux often promotes cell death. In autophagy, microtubule-associated protein 1-light chain 3 (LC3) is dissociated, lipidated, and converted to LC3-II, which then moves to the autophagosome membrane. High levels of LC3-II proteins and LC3-II-enclosed autophagosomes are critical markers of autophagy (73, 74). Findings from this study showed that oesophageal CSCs subjected to AlPcS_4_Cl and AlPcS_4_Cl-AuNP-based PDT had a negligible effect on LC3-II formation, demonstrating no autophagy induction. Hence, AlPcS_4_Cl and AlPcS_4_Cl-AuNP-mediated PDT do not promote autophagy cell death in oesophageal CSCs. This report aligns with our previous study on oesophageal cancer cells (33). Conversely, findings on NIC-based PDT cells were shown to induce autophagy in oesophageal CSCs, as demonstrated by the increased expression of LC3-II proteins. The induced autophagy observed in this study may possibly be due to the anti-CD271 antibody drug conjugates, as this aligns with previous study by wang and co-worker where antibody drug conjugate rituximab-monomethyl auristatin E was found to induce autophagy (75). However, a study by Lin and colleagues (76) showed that pH-sensitive polymeric nanoparticles with gold (I) compound simultaneously induced apoptosis and autophagy. In summary the findings from this study suggest that the NIC-mediated PDT induced dual cell death mechanisms via apoptosis and autophagy in oesophageal CSCs **Figure 12**. 

## 5 Conclusion

Oesophageal cancer is ranked as the eleventh cause of cancer-associated sickness and the seventh cancer-related death globally (2). Currently, conventional therapy is insufficient in treating this disease, and advancements in medicine have been unable to conquer it. This failure is thought to be due to CSCs. CSCs are fortified with different mechanisms such as self-renewal potential, aggressive proliferation, metastasis, unique surface markers, and the potential to escape treatment pressure due to drug-efflux membrane proteins and the expression of high levels of anti-apoptotic proteins. Therefore, enhanced delivery of anti-tumour agents aimed at targeting CSCs offers a proactive and efficient treatment measure to eradicate CSCs and improve anti-tumour drug delivery. In this study, we analysed the effects of nano-immuno-targeted PDT on oesophageal cancer stem cells and associated cell death mechanisms. The nano-immuno-targeted PDT is a combination of AlPcS_4_Cl, AuNPs, and anti-CD271 antibody to form the AlPcS_4_Cl-AuNP-anti-CD271 antibody conjugates, known as NIC (nano-immuno-conjugates), which are of nanosized in diameter. The NIC-mediated PDT demonstrated improved anti-tumour features such as improved antiproliferative effects, increased cytotoxicity, high ROS production, and ΔΨM disruption. Damage to the mitochondrial membrane was validated by the efflux of cytochrome c and the increased activation of caspase 3/7 enzyme observed in our study. Our findings also demonstrated the impacts of NIC-based PDT in promoting DNA damage through the ATM and DNA DSB response pathways and the induction of G0/G1 cell cycle arrest on oesophageal CSCs.

Furthermore, our investigation of the cell death mechanism showed that NIC-mediated PDT initiated two conventional cell death pathways: apoptosis and autophagy. Autophagy was probably triggered as a pro-survival action of the CSCs to escape apoptosis. The induction of autophagy was established through the elevated levels of LC3 protein, a potent indicator of autophagic cell death (77, 78). Further investigations are required to validate this finding. Overall, this study demonstrated the cell death mechanisms and the promising application of gold nano-immunoconjugate-based PDT in eradicating oesophageal CSCs and oesophageal cancer. However, the absence of NIC-mediated PDT effects on normal oesophageal cells presents a limitation to this study. The impacts of NIC-mediated PDT on normal oesophageal cells and different cancer cells should be confirmed. Also, this study is an *in vitro* model, which does not reflect the true nature of an *in vivo* model, in which other factors might influence the outcome. Normal cells in the body expressing similar receptors of interest (CD271) and the tumour microenvironment may impact the outcome of this treatment. Therefore, further evaluations are needed to assess the impact of NIC-mediated PDT on oesophageal cancer in an *in vivo* preclinical model. Finally, the effects of NIC-mediated PDT on apoptosis gene expression profile, various signalling pathways, and transcriptional factors that drive the pro-survival mechanism of oesophageal CSCs are warranted.

## Data Availability

The original contributions presented in the study are included in the article/**Supplementary Material**. Further inquiries can be directed to the corresponding author.
