# Supplementary material for: Cell death mechanisms induced by gold nano-immunoconjugates-mediated photodynamic therapy against human oesophageal cancer stem cells
Source: Front Immunol. 2025 Sep 16;16:1585251. doi: 10.3389/fimmu.2025.1585251 (PMC12479469; doi:10.3389/fimmu.2025.1585251)
Supplement: Supplementary file 1 [file Table1.docx]

**Physicochemical Characterisation of the Nano-Immuno-Construct (NIC) Photosensitizer**

## 1. UV-vis spectroscopy and loading efficiency


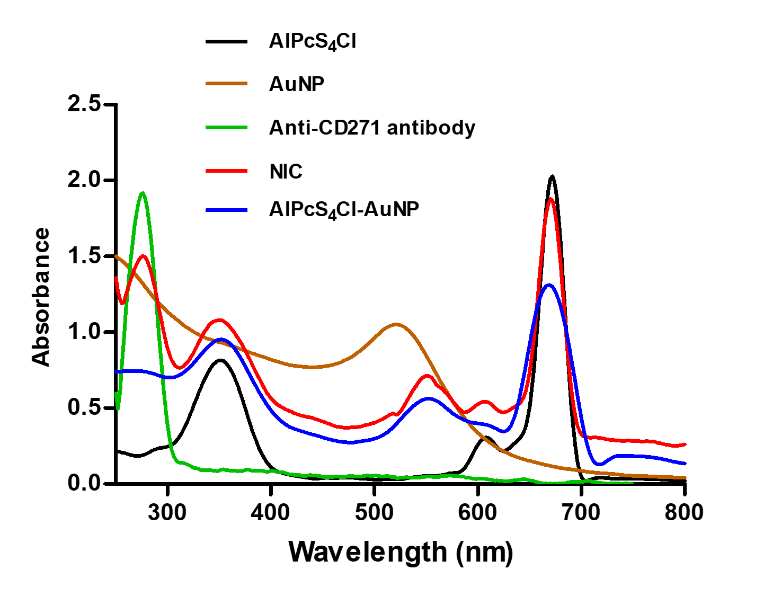


**Figure 1: UV-Vis absorption spectra analysis of NIC, APcS4Cl-AuNPs, AuNPs, Anti-CD271 antibody and AlPcS4Cl. The various absorption spectral peaks were noted with 278 nm for the Anti-CD271 antibody, 520 nm and 542 nm for AuNP, and 350 nm, 674 nm, 672 nm and 668nm for AlPcS4Cl.**


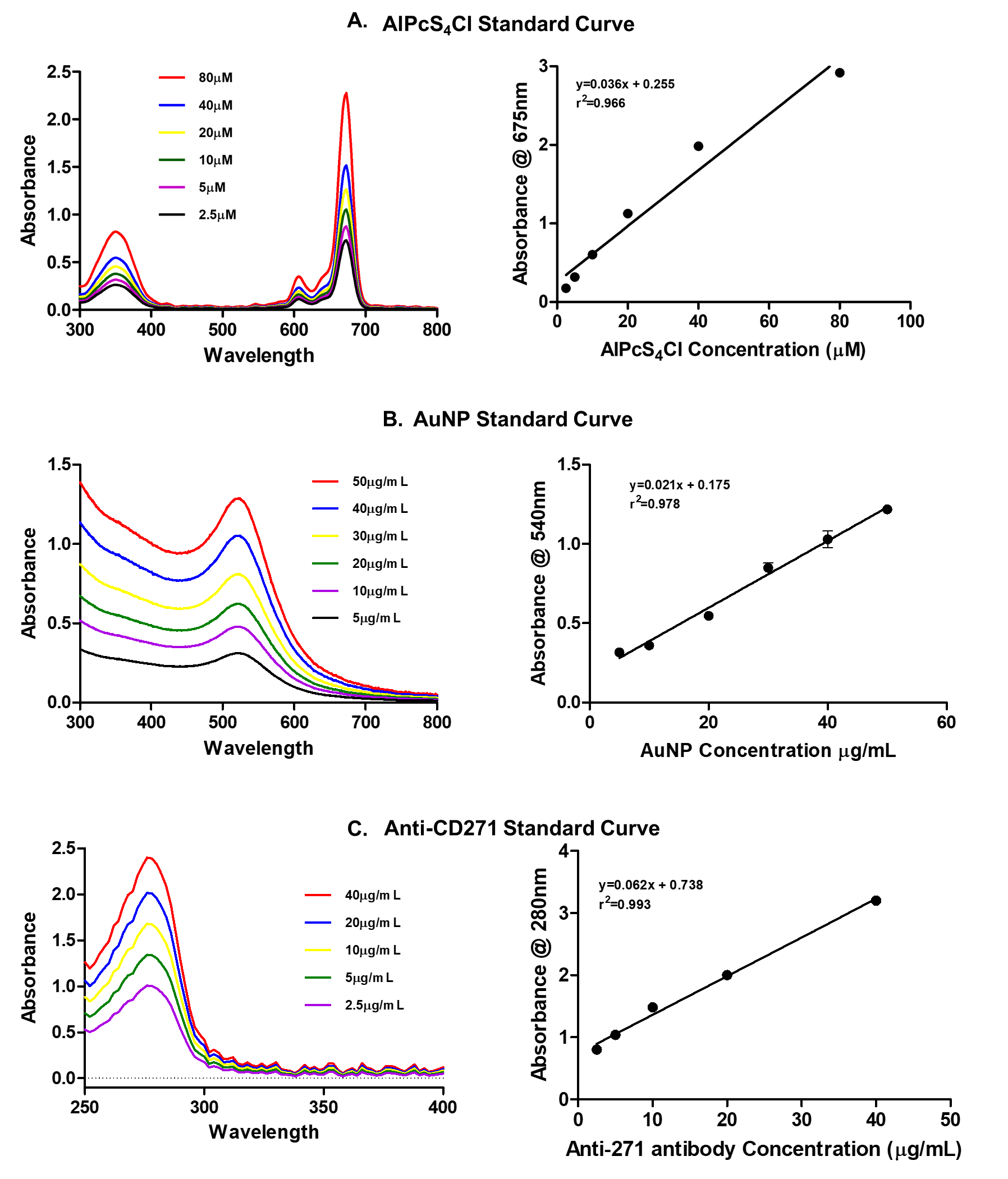


**Figure 2: The UV-Vis absorption spectra and the standard calibration curve corresponding of AlPcS4Cl (A), AuNPs (B) and Anti-CD271 antibody (C) demonstrated linearity.**

$$Conjugation efficiency \left( \% \right)=(Final drug concentration\div Total drug concentration added)\times100$$

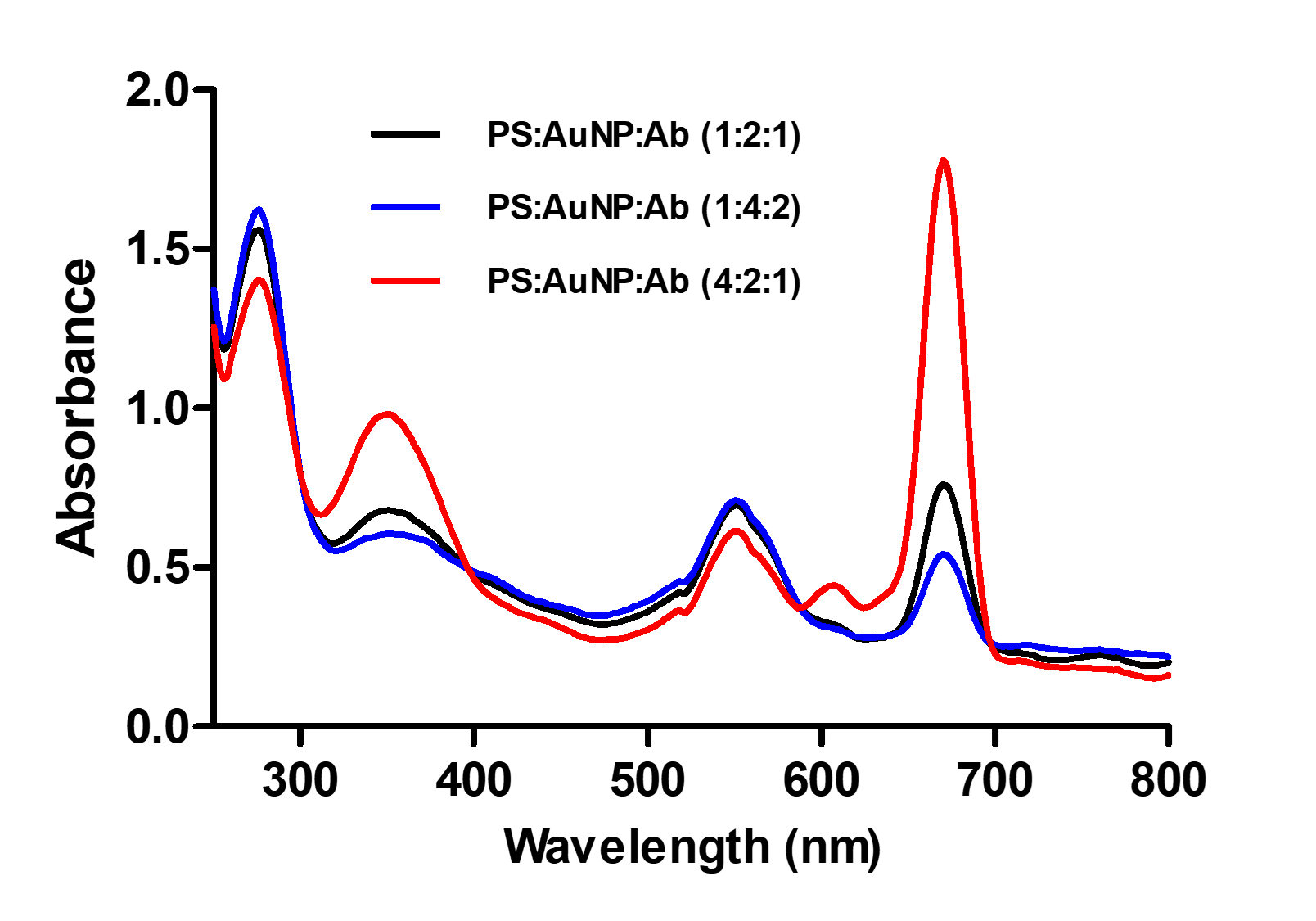


**Figure 3: The UV-Vis absorption spectra analysis of NIC with the various AlPcS4Cl/AuNP/Anti-CD271 ratios used in NIC preparation.**

**Table 1: AlPcS4Cl/AuNP/Anti-CD271 ratio used in NIC preparation and the conjugation loading efficiency of the individual component in the NIC.**

| AlPcS_4_Cl/AuNP/Anti-CD271 Ratio | Absorbance | Formula | Total  Concentration  added | Concentration  In the NIC | Loading  Efficiency (%) |
| --- | --- | --- | --- | --- | --- |
| AlPcS_4_Cl | 675 nm | y = 0.036x + 0.255 |  |  |  |
| 1:2:1 | 0.82 |  | 20 µM | 15.5 µM | 77.5 |
| 1:4:2 | 0.51 |  | 10 µM | 6.9 µM | 69.0 |
| 4:2:1 | 1.95 |  | 80 µM | 48.6 µM | 60.8 |
| AuNP | 540 nm | y = 0.021x + 0.175 |  |  |  |
| 1:2:1 | 0.76 |  | 40 µg/mL | 27.9 µg/mL | 69.8 |
| 1:4:2 | 0.70 |  | 40 µg/mL | 25.0 µ/mL | 62.5 |
| 4:2:1 | 0.69 |  | 40 µg/mL | 24.5 µg/mL | 61.3 |
| Ant-CD271 antibody | 280 nm | y= 0.062x + 0.738 |  |  |  |
| 1:2:1 | 1.60 |  | 20 µg/mL | 13.9 µg/mL | 69.5 |
| 1:4:2 | 1.66 |  | 20 µg/mL | 14.8 µg/mL | 74.0 |
| 4:2:1 | 1.44 |  | 20 µg/mL | 11.2 µg/mL | 56.0 |

## 2. TEM particle size analysis


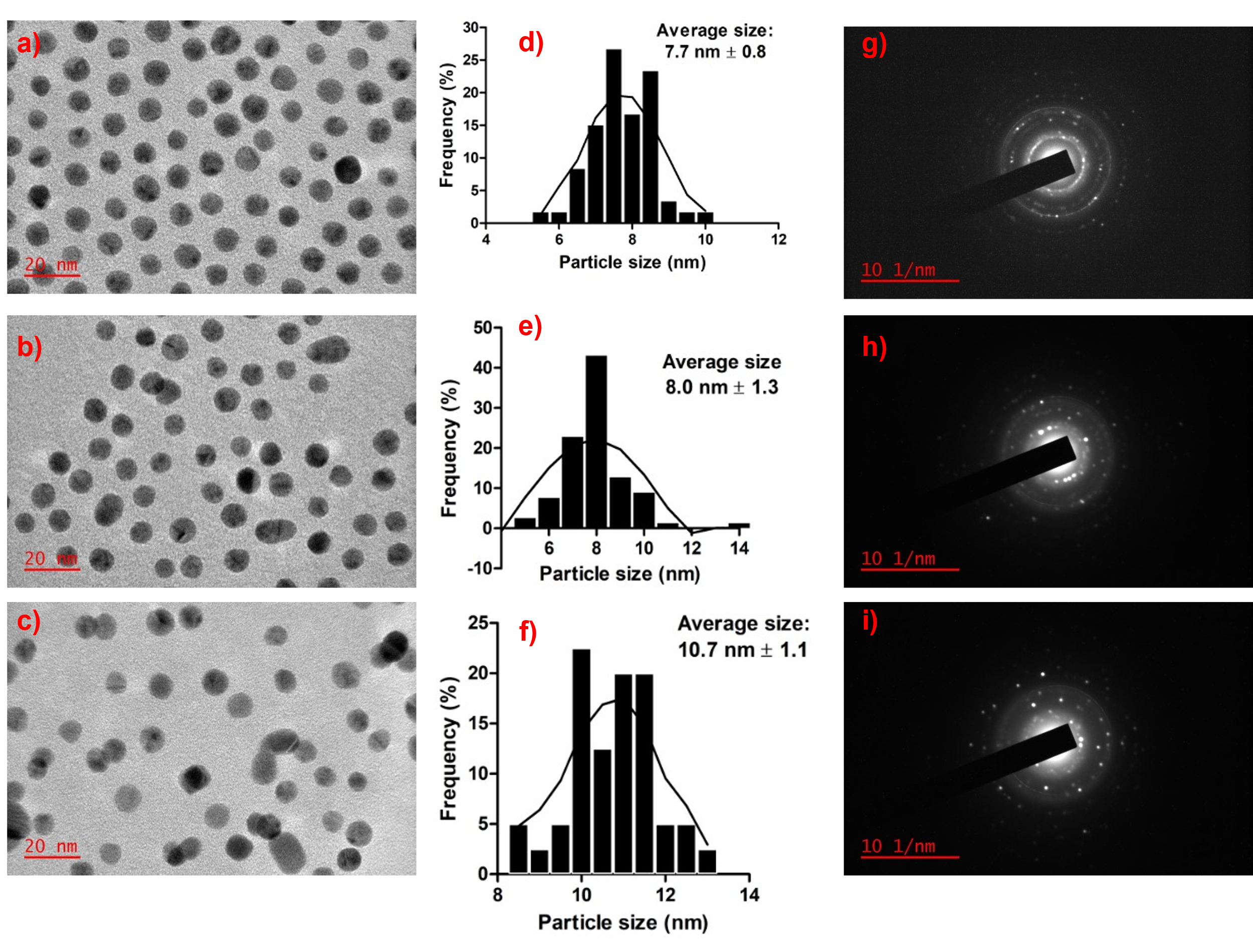


**Figure 4: TEM analysis displaying the morphological features of a) AuNP, b) AlPcS4Cl-AuNPs and c) NIC. Images are representative of three captured fields. Histogram of the nanoparticle size distribution of d) AuNP, e) AlPcS4Cl-AuNPs and f) NIC. The SAED pattern for g) AuNP, h) AlPcS4Cl-AuNPs and i) NIC.**

## 3. FTIR spectroscopy

**
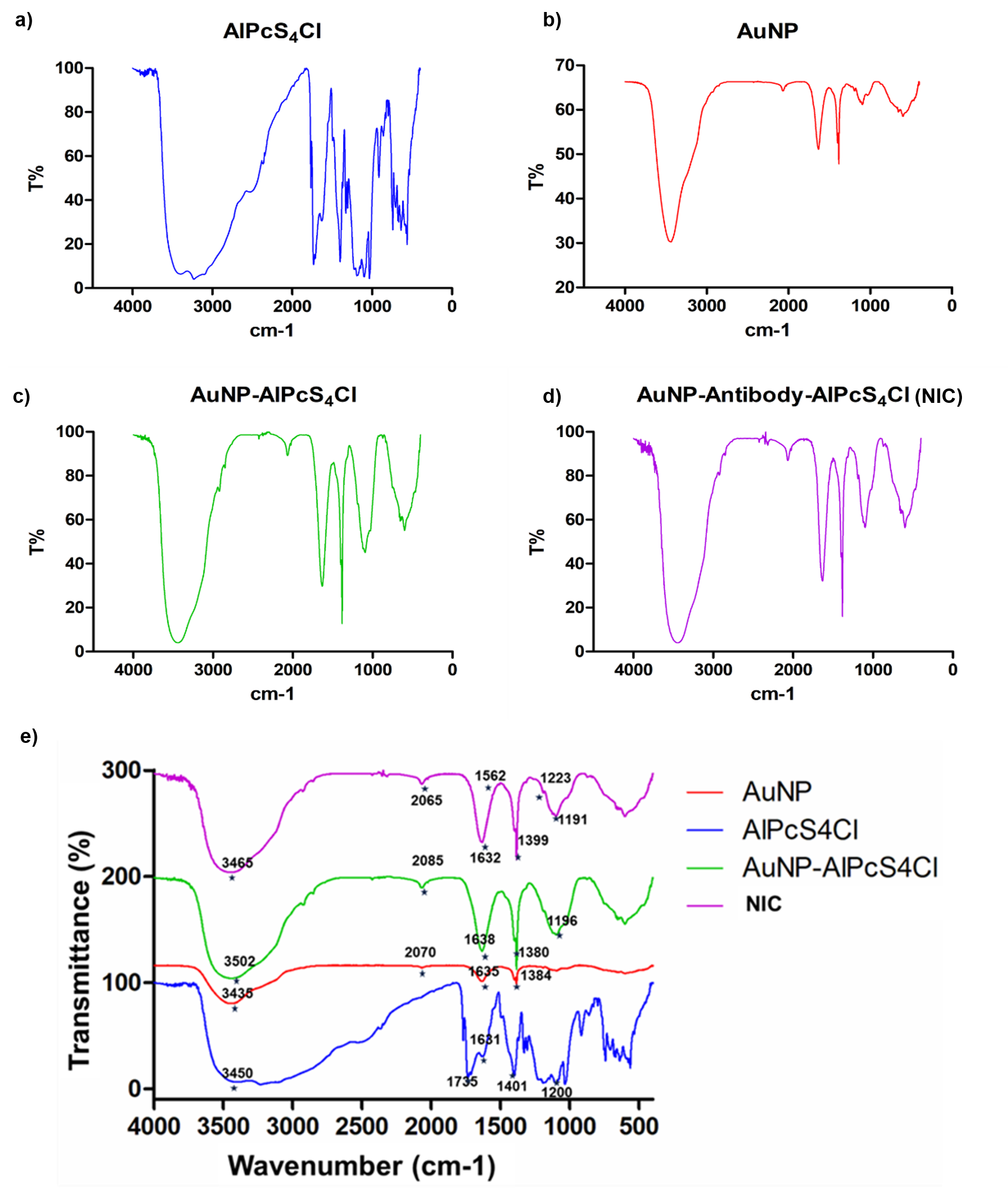
**

**Figure 5: FTIR spectra showing the chemical features of AlPcS4Cl (a), AuNPs (b), AlPcS4Cl-AuNP (c), AlPcS4Cl-AuNP-Antibody (NIC) (d) and the combined FTIR (e).**

## 4. Dynamic light scattering and zeta potential evaluation

**Table 2: Dynamic light scattering (DLS)** **polydispersity index (PdI) and Zeta potential of the unconjugated AuNP suspension, AlPcS4Cl-AuNP and NIC.**

| Agents | DLS (d. nm) | PdI | Zeta Potential |
| --- | --- | --- | --- |
| AuNP | 37.71 ± 1.69 | 0.351 ± 0.01 | -10.90 ± 1.21 |
| AlPcS_4_Cl- AuNP | 169.7 ± 21.36 | 0.460 ± 0.02 | -0.923 ± 0.25 |
| NIC | 467.9 ± 12.89 | 0.557 ± 0.09 | -12.1 ± 0.20 |
